# Decoding everyday levels of musical training from subcortical white-matter architecture

**DOI:** 10.1162/IMAG.a.1325

**Published:** 2026-08-04

**Authors:** Massimo Lumaca, Marcus T. Pearce, Peter E. Keller, Peter Vuust, Elvira Brattico, Giosuè Baggio, Katarzyna Hat, Ole A. Heggli, Kristian Sandberg

**Affiliations:** Center for Music in the Brain, Department of Clinical Medicine, Aarhus University & The Royal Academy of Music Aarhus/Aalborg, Aarhus, Denmark; School of Electronic Engineering and Computer Science, Queen Mary University of London, London, United Kingdom; The MARCS Institute for Brain, Behaviour and Development, Western Sydney University, Penrith, Australia; Department of Education, Psychology, Communication, University of Bari, Bari, Italy; Language Acquisition and Language Processing Lab, Norwegian University of Science and Technology, Trondheim, Norway; Consciousness Lab, Institute of Psychology, Jagiellonian University, Kraków, Poland; Center of Functionally Integrative Neuroscience, Department of Clinical Medicine, Aarhus University, Aarhus, Denmark; Neurobiology Research Unit, Copenhagen University Hospital Rigshospitalet, Copenhagen, Denmark

**Keywords:** network neuroscience, machine learning, musical training, neuroplasticity, subcortical circuits, non-musicians

## Abstract

Most people engage with music informally or through the standard school curriculum rather than through extensive professional practice, resulting in graded levels of accumulated musical training. In this study, we used cross-validated machine learning on whole-brain structural connectomes to investigate whether individual differences in brain wiring predict variation in musical training across 225 adults with none-to-moderate training levels. Connectomes were weighted by fiber bundle capacity (FBC), a quantitative diffusion MRI metric of structural connectivity. Musical training was quantified using the Gold-MSI Musical Training subscale (F3), a self-reported measure encompassing both formal and informal musical practice. Subcortical white-matter pathways linking thalamus, putamen, and pallidum with sensorimotor cortex carried the strongest predictive signal (r = 0.261; R^2^ = 0.068; p_perm = 0.003). Furthermore, this connectivity predicted the association between musical training and auditory perception skill, which was present in individuals with stronger subcortical–sensorimotor connectivity (+1 SD: β = 0.192, p = 0.004), but absent in those with weaker connectivity (−1 SD: β = −0.066, p = 0.488). Subcortical–sensorimotor connectivity is, therefore, the strongest structural correlate of graded musical training in non-specialists within the general population, identifying the structural neural substrate on which the training–skill association depends. This work warrants renewed targeted focus on subcortical circuits in models of musical expertise.

## 1 Introduction

Individuals who invest comparable time and effort in music often attain markedly different skill levels (Ericsson et al., 1993; Hallam, 1998; Sloboda et al., 1996; Sosniak, 1990). Understanding the neural basis of this variability is central to identifying neural markers of learning potential. While machine learning (ML) is well suited to uncover such markers (Gabrieli et al., 2015), its application to neuroimaging data has traditionally relied on expert–novice binary classification (Grecucci et al., 2023; RaviPrakash et al., 2021; Saari et al., 2018; Shim et al., 2021), which does not capture individual variability and the continuous spectrum of training experience typical of the general population (Jimenez & Elliott, 2024; Müllensiefen et al., 2014). This binary approach also introduces a key confound: experts and novices often differ in both accumulated training and aptitude, making it difficult to separate these factors statistically in neural signatures of expertise (Correia et al., 2023). To address these limitations, we applied prediction-based ML to musical training—a well-established model system of neuroplasticity (Herholz & Zatorre, 2012; Münte et al., 2002; Wan & Schlaug, 2010) that exhibits continuous variation across non-specialists (Jimenez & Elliott, 2024; Müllensiefen et al., 2014) and can be measured with validated psychometric instruments (Müllensiefen et al., 2013).

Neuroimaging studies of musical expertise have largely focused on structural brain changes associated with intensive, prolonged musical training in adults (Criscuolo et al., 2022; Münte et al., 2002). Typically, these studies compare highly skilled musicians with untrained listeners (so-called non-musicians), with expertise operationalized categorically through years of formal instruction above an arbitrary threshold—for example, 5, 6, or 10 years (Daly & Hall, 2018; Dowling et al., 1995; J. D. Zhang et al., 2020). This work has consistently revealed large-scale structural differences between groups, primarily within sensorimotor and executive-control networks (Criscuolo et al., 2022; Penhune, 2019). These adaptations include changes in gray-matter morphology (Abdul-Kareem et al., 2011; Bailey et al., 2014; Bermudez et al., 2009; Foster & Zatorre, 2010; Gaser & Schlaug, 2003; Hutchinson et al., 2003; James et al., 2014; Schneider et al., 2005; Vaquero et al., 2016) and white-matter architecture (Bengtsson et al., 2005; Halwani et al., 2011; X. Li et al., 2021; Moore et al., 2014; Oechslin et al., 2009; Schmithorst & Wilke, 2002; Steele et al., 2013). This literature showed a predominant “cortico-centric” focus. As detailed in Supplementary Table S1, the majority of diffusion MRI studies (Mori & Zhang, 2006) looking at expertise-related white-matter adaptations have deliberately targeted, through a priori region-of-interest analyses, tracts connecting cortical regions: the corpus callosum (Burunat et al., 2017; Elmer et al., 2016; Habibi et al., 2018; Loui et al., 2019; Schlaug et al., 1995; Steele et al., 2013), the arcuate fasciculus (Halwani et al., 2011; X. Li et al., 2021; Vaquero et al., 2018, 2021), and the superior longitudinal fasciculus (Loui et al., 2019; Oechslin et al., 2009). Cortico-subcortical tracts have received far less targeted attention: descending motor pathways such as the corticospinal tract have been examined as primary ROIs in only a handful of studies (Engel et al., 2014; Giacosa et al., 2019; Imfeld et al., 2009; Rüber et al., 2015), with inconsistencies reported across studies in FA directions (Moore et al., 2014; Schlaug, 2015): lower for musicians in some (Imfeld et al., 2009; Schmithorst & Wilke, 2002) and higher in others (Bengtsson et al., 2005; Han et al., 2009; Rüber et al., 2015). Subcortical structures (thalamus, putamen, cerebellum) also show morphological differences (Gaser & Schlaug, 2003; Hutchinson et al., 2003; Vaquero et al., 2016), but their long-range connectivity patterns and relationship to individual variability in skill acquisition remain less explored. Nevertheless, these studies provide robust evidence that extensive practice is associated with large-scale changes in brain structure and that magnetic resonance imaging (MRI) can detect neural signatures of expertise. However, it remains unresolved whether these signatures extend to the graded variation in training observed in adults with little or no musical training (Lappe et al., 2011) and whether observed brain–behavior associations primarily reflect accumulated experience, predispositions, third variables, or their combination.

Historically, studies have treated non-musicians as a uniform comparison group, conflating the absence of formal instruction with low musical ability (Bigand & Poulin-Charronnat, 2006; Correia et al., 2023; Law & Zentner, 2012). In reality, untrained listeners have vast differences in musical experience and how deeply they engage with music (Müllensiefen et al., 2014). Also, they span a wide range of musical ability shaped by genetic predispositions and informal musical experience (Folkestad, 2006; Gingras et al., 2015; Mosing et al., 2014; Tan et al., 2014; Veblen, 2018), with some individuals possessing perceptual expertise (‘listening expertise’) comparable with that of trained musicians (Correia et al., 2023). Informal engagement can refine perceptual skills through everyday musical activities (e.g., listening, singing, and dancing), even without formal instruction (Bigand & Poulin-Charronnat, 2006). Rather than being a nuisance, this heterogeneity offers a methodological opportunity to disentangle the effects of training and aptitude. In expert–novice comparisons, training is tightly coupled to pre-existing ability, making it difficult to separate experience-dependent plasticity from predisposition (Schellenberg, 2020). Because professional musicians typically possess both high perceptual abilities and extensive training, observed neural differences may reflect both pre-existing biological markers of musicality and plasticity (Miendlarzewska & Trost, 2013). In contrast, in non-musicians, these factors can be partially decoupled: high aptitude can occur with low or modest training, and training can accumulate with low or modest aptitude (Correia et al., 2023; Swaminathan & Schellenberg, 2018). This partial decoupling allows training and aptitude to be examined as partially independent sources of variation in brain structure.

We leverage this partial dissociation by measuring training with the Goldsmiths Musical Sophistication Index (Gold-MSI) Musical Training subscale (Factor 3, hereafter “F3”) (Müllensiefen et al., 2013) and perceptual ability with the Musical Ear Test (MET) (Wallentin et al., 2010). Both instruments have been validated in large-scale studies involving listeners from different countries and cultural backgrounds (Correia et al., 2022; Klarlund et al., 2023; J. Li et al., 2024; Müllensiefen et al., 2014; Swaminathan et al., 2021; Wang et al., 2024). F3 is a continuous measure spanning minimal engagement to formal instruction that correlates with objective measures of musical ability (Correia et al., 2022, 2023; Lumaca et al., 2024; Müllensiefen et al., 2014). The MET provides a continuous measure of auditory perceptual ability that can vary independently of formal training. Detecting training-related neural signatures in this population is challenging because the effects of short-term training typical of ordinary musical education are subtler than those observed in expert–novice contrasts (Dinse, 2021). Reliable detection requires large samples (often N > 100) and out-of-sample validation procedures (Boekel et al., 2015; Button et al., 2013; Gabrieli et al., 2015; Poldrack et al., 2017). Moreover, plasticity likely produces high-dimensional, distributed effects in network architecture (Leipold et al., 2021), necessitating multivariate approaches sensitive to small differences in network-level reorganization across individuals (Fornito et al., 2016; Jeon & Friederici, 2017).

To address these challenges, we used a prediction-based network neuroscience approach called NBS-Predict (Serin et al., 2021), which extends Network-Based Statistics (NBS) (Zalesky et al., 2010) from group-level inference to single-subject prediction. NBS-Predict was applied to musical training as a continuous measure in a large sample of adults with little or no formal musical training. Our predictors were whole-brain structural connectivity matrices (maps of white-matter connections across the brain) derived from high-resolution diffusion MRI (Descoteaux, 2015) and anatomical images (Weiskopf et al., 2013), and weighted by fiber bundle capacity (FBC), a quantitative diffusion MRI metric that estimates white-matter connectivity strength (Smith et al., 2022). NBS-Predict identifies predictive subnetworks and weights each edge by its out-of-sample contribution. Beyond prediction, we tested whether connectivity in the most predictive network was statistically associated with the relationship between musical training (Gold-MSI F3) and auditory perceptual skill (MET) (Kraus & Chandrasekaran, 2010). To examine this question, we employed cross-sectional mediation and moderation analyses. These statistical models cannot distinguish between training-related plasticity, genetic, or other pre-existing factors. However, they can address two more specific questions. First, the mediation model tests whether participants with higher levels of musical training also tend to exhibit stronger connectivity, and whether stronger connectivity is, in turn, associated with better perceptual skill. Second, the moderation model tests whether the association between musical training and perceptual skill is consistent across participants or instead varies as a function of connectivity strength. In the present study, these models indicated that the training–perception association was not uniform but varied with subcortical–cortical connectivity strength, being only present in individuals with stronger connectivity. These analyses may help guide future longitudinal or genetically informed studies by indicating whether such conditional associations with connectivity mainly reflect experiential, genetic, or other factors.

## 2 Methods

### 2.1 Participants

Data were collected as part of the EU COST Action CA18106 *The Neural Architecture of Consciousness* across multiple sessions at Aarhus University and Aarhus University Hospital. The study was approved by *De Videnskabsetiske Komitéer for Region Midtjylland*, Denmark (Project ID: M-2016-69-16). All participants gave informed consent and were financially compensated.

Three hundred healthy adults (aged 18–49 years), with no history of neurological or psychiatric disorders and no reported hearing deficits, were recruited through the Center of Functionally Integrative Neuroscience (CFIN) at Aarhus University using the university’s participant database and local advertisements. All participants completed the MRI scanning session and the Goldsmiths Musical Sophistication Index (Gold-MSI) questionnaire, both mandatory components. Of these, 241 participants (135 female) completed the optional Musical Ear Test (MET). Multivariate outlier detection (using PCA and Euclidean distance) identified nine participants with extreme Gold-MSI and MET profiles, who were excluded from further analysis (Supplementary Fig. S1). An additional 7 participants were excluded due to failure in structural network reconstruction, yielding a final sample of 225 participants with Gold-MSI and MET data. Self-reported musical training showed that approximately 47% (N = 105) had up to 5 years of training, and only approximately 13% (N = 31) had more than 6 years (J. D. Zhang et al., 2020) (Supplementary Fig. S2). Three reported formal music education at university level: one trained as a saxophone soloist at a conservatory, one held a bachelor’s degree in rhythmic music, and one in musicology and religious studies. Of the final sample, 222 participants also completed the *Wechsler Adult Intelligence Scale – Fourth Edition (WAIS-IV)* (Lichtenberger & Kaufman, 2009).

### 2.2 Musical training and perceptual ability

#### 2.2.1 Goldsmiths Musical Sophistication Index (Gold-MSI)

The Gold-MSI is a self-report inventory measuring individual differences in musical behavior, experience, and skills in the general or “non-specialist” population. It comprises 38 items across 5 subscales: Active Engagement, Perceptual Abilities, Music Training, Singing Abilities, and Emotions. A General Musical Sophistication factor is derived from 18 items across these subscales. Items 1–31 are rated on a 7-point Likert scale; the remaining 7 use variable formats. Subscale scores were computed using the official Gold-MSI Scorer App (https://shiny.gold-msi.org/gmsiscorer).

This study focused on the Music Training subscale (Factor 3, hereafter F3), a multidimensional index of formal and informal musical experience comprising seven items: years of formal instruction, music theory training, hours of peak practice, instruments played, years of active engagement, self-identification as a musician, and frequency of receiving compliments on musical skill.

#### 2.2.2 Musical Ear Test (MET)

The MET is a 104-trial test of musical perceptual ability (Wallentin et al., 2010), split evenly between melodic (52 piano-tone pairs) and rhythmic (52 drum-beat pairs) subtests. Participants judged whether the sequences in each pair were identical or different; deviations involved a single tone (Melody) or inter-onset interval (Rhythm). Instructions emphasized headphone use and a distraction-free environment, and feedback was given only during practice. Scores reflected the percentage of correct responses per subtest and total (out of 104).

Given its high correlation with both Melody (r = 0.89) and Rhythm (r = 0.85) scores (Supplementary Fig. S4), the MET total score served as the primary measure of musical perceptual ability.

### 2.3 General cognitive abilities

#### 2.3.1 Wechsler Adult Intelligence Scale (WAIS)

The WAIS-IV is a standardized assessment of adult intellectual functioning, comprising 10 core subtests that yield 4 index scores-Verbal Comprehension, Perceptual Reasoning, Working Memory, and Processing Speed-and a Full-Scale IQ (FSIQ) composite (M = 100, SD = 15). Full-scale IQ served as a covariate in subsequent mediation and moderation analyses.

### 2.4 MRI acquisition

Data were acquired on a Siemens Magnetom Prisma-fit 3T MRI scanner with a 32-channel head coil during a 1-hour session comprising two resting-state fMRI scans (12 and 6 minutes), quantitative multi-parameter mapping (MPM) (Weiskopf et al., 2013)—used here for synthetically generated T1-weighted images (20 minutes)—and high-angular resolution diffusion imaging (HARDI) (10 minutes). Only MPM-derived anatomical and HARDI images were used in this study.

The MPM protocol consisted of three multi-echo 3D FLASH sequences (MT-, PD-, and T1-weighted), plus reference RF scans, following a Siemens vendor protocol. Parameters: TR = 18 ms (PD- and T1-weighted) and 37 ms (MT-weighted); minimum/maximum TE = 2.46/14.76 ms; flip angles = 6° (MT), 4° (PD), 25° (T1); six equidistant echoes; voxel size = 1 mm³ isotropic; field of view = 224 × 256 × 176 mm; AP phase encoding; GRAPPA factor = 2. Radiofrequency field inhomogeneity was characterized with two reference maps: a transmit field (B1^+^) map (TR = 2,000 ms; TE = 14 ms; matrix = 64 × 64 × 18; 4 × 4 × 10 mm) and receive field (B1^−^) sensitivity maps estimated from low-resolution 3D gradient-echo volumes acquired with both the 32-channel head coil and the body coil (TR = 3.8 ms; TE = 1.71 ms; 4 mm isotropic; matrix = 56 × 64 × 44), acquired before each of the T1-, PD-, and MT-weighted contrasts (Leutritz et al., 2020; Papp et al., 2016; Tabelow et al., 2019).

HARDI used a multiband multi-shell echo-planar sequence with 211 directions across six b-shells (b = 5, 700, 1000, 1200, 1500, and 2500 s/mm^2^, with 10, 15, 30, 21, 60, and 75 directions, respectively). This b-value sampling was optimized for DTI and DKI estimation rather than for fiber bundle capacity (FBC) per se. Parameters: TR = 3,850 ms; TE = 71 ms; flip angle = 90 degrees; voxel size = 2 mm^3^; matrix = 100 x 100; 84 axial slices; anterior-to-posterior (AP) phase encoding; multiband (simultaneous multi-slice) acceleration factor = 3; in-plane GRAPPA acceleration factor = 2. Five additional b = 0 s/mm^2^ volumes in the reverse (PA) direction were acquired for EPI distortion correction (Andersson et al., 2003).

### 2.5 Creation of synthetic T1-weighted images

Synthetic T1-weighted images were generated from high-resolution longitudinal relaxation rate (R1) and effective proton density (PD) maps acquired via MPM. The R1 map was converted to T1 by taking its reciprocal, thresholded at zero, and scaled by 1,000; the PD map was thresholded at 0 and scaled by 100 (using FSL fslmaths). Freesurfer mri_synthesize then generated synthetic FLASH images from these maps using weighting parameters (20, 30, 2.5) to enhance gray–white-matter contrast, with a final intensity rescaling (division by 4) to match FreeSurfer conventions.

Synthetic T1-weighted images were preprocessed using fMRIPrep 21.0.2 (Esteban et al., 2019) (RRID:SCR_016216), based on Nipype 1.6.1 (Gorgolewski et al., 2011). Steps included intensity non-uniformity correction (N4BiasFieldCorrection (Tustison et al., 2010), ANTs 2.3.3 (Avants et al., 2008)), skull stripping (antsBrainExtraction.sh, targeting the OASIS30ANTs template), and tissue segmentation of cerebrospinal fluid (CSF), white matter (WM), and gray matter (GM) using FAST (Y. Zhang et al., 2001) (FSL 6.0.5.1; RRID:SCR_002823)

Cortical surface reconstruction used FreeSurfer’s recon-all (v6.0.1; RRID:SCR_001847) (Dale et al., 1999), with a custom Mindboggle variation (A. Klein et al., 2017) (RRID:SCR_002438) to reconcile ANTs-derived and FreeSurfer-derived segmentations of cortical GM. Volume-based spatial normalization to MNI space used antsRegistration (ANTs 2.3.3) with the ICBM 152 Nonlinear Asymmetrical 2009c template (RRID:SCR_008796) and FSL’s MNI ICBM 152 Nonlinear 6th Generation template (Evans et al., 2012).

### 2.6 dMRI preprocessing and structural brain network construction

Diffusion MRI (dMRI) data were preprocessed using custom MATLAB scripts developed at the CFIN. Preprocessing included noise reduction (Veraart et al., 2016), Gibbs ringing artifact correction (Kellner et al., 2016), and correction for motion, eddy currents, and susceptibility-induced distortions using topup and eddy from FSL (Andersson & Sotiropoulos, 2016). The b = 5 s/mm^2^ volumes, whose diffusion weighting is negligible, were treated as b = 0 during topup and eddy correction.

Structural connectivity matrices were constructed using the MRtrix3 (Tournier et al., 2019). For each participant, a five-tissue-type image was created, segmenting cortical gray matter, deep gray matter, white matter, CSF, and “other” tissues—required for Anatomically-Constrained Tractography (ACT) (Smith et al., 2012). Synthetic T1-weighted anatomical images were co-registered to diffusion space, and tissue-specific response functions (white matter, gray matter, CSF) were estimated individually and averaged to generate group-level response functions. Multi-Shell Multi-Tissue Constrained Spherical Deconvolution estimated fiber orientation distributions within each voxel, followed by intensity normalization (Jeurissen et al., 2014).

Whole-brain probabilistic tractography used ACT (Anatomically Constrained Tractography) with dynamic seeding and backtracking. Up to 1 × 10^9^ streamlines were attempted, from which 10 million were selected per participant. Seeding was dynamically determined from the fiber orientation distribution (FOD) image following the Spherical-deconvolution Informed Filtering of Tractograms (SIFT) model (Smith et al., 2013). Tractography parameters included an FOD amplitude cutoff of 0.06, minimum streamline length of 20 mm, and maximum of 250 mm. The SIFT2 algorithm filtered the tractogram from negligible connection weights (Smith et al., 2015), estimating fiber bundle capacity (FBC) of white-matter connections. FBC estimates the total intra-axonal cross-sectional area by summing SIFT2-weighted streamlines and scaling by a participant-specific proportionality coefficient (mu) that corrects for biases in seeding density and tract length (Smith et al., 2022). Unlike fractional anisotropy or streamline count, FBC provides an interpretable proxy of information-carrying capacity, or the “bandwidth” of a bundle. The FBC formula is

$${\text{FBC}}_{p,i}\;=\mu_{i}\sum_{s_{i}\in S_{p}}w_{s_{i}},$$

where FBC_p,i_ is the capacity of pathway *p* for subject *i*, μ is the participant-specific proportionality coefficient, w_si_ is the weight assigned to streamline s_i_, S_p_ is the set of streamlines belonging to pathway *p*. FBC-weighted structural connectivity matrices were constructed by combining the cortical Destrieux parcellation (148 cortical regions; 74 per hemisphere) with subcortical segmentations from FSL FIRST tool (16 subcortical regions; 8 per hemisphere, excluding brainstem), yielding 164 × 164 whole-brain matrices. The resulting networks contained 13,312 unique edges (99.6% of the theoretical maximum of 13,366 possible connections). A custom automated pipeline performed quality control, generating standardized visualizations of processed diffusion data, including static JPEGs of five-tissue-type segmentation and animated GIFs of T1w-to-b0 co-registration, which were visually inspected.

### 2.7 Log-transformation of whole-brain FBC matrices

FBC-weighted structural matrices exhibit extreme right-skewed distributions (Supplementary Fig. S3) with many weak connections and a small subset of disproportionately strong tracts. This skewness impairs machine learning (ML) performance by allowing high-magnitude features to dominate distance-based calculations and model optimization. Following recent practices (X. Li et al., 2023; Sangchooli et al., 2025), logarithmic transformation was applied to FBC values before ML analyses with NBS-Predict.

### 2.8 NBS-Predict pipeline

Whole-brain (log)FBC matrices were analyzed using NBS-Predict (www.nitrc.org/projects/nbspredict), a multivariate ML pipeline integrating network-based feature selection with predictive modeling under a cross-validation framework. Repeated 10-fold nested cross-validation (10 repetitions; 100 train–test partitions) ensured robust generalizability. The outer loop assessed model performance on held-out data; the inner loop performed grid search (5 steps) to optimize hyperparameters. Within each training fold, a general linear model (GLM) was fitted to each edge, with FBC as the dependent variable and F3, age, biological sex, and estimated intracranial volume (eITV) as predictors, using a threshold of p < 0.01 (Serin et al., 2021). A breadth-first search (BFS) algorithm (Hopcroft & Tarjan, 1973) identified the largest connected (graph) component among suprathreshold edges, constraining model input to biologically plausible subnetworks rather than isolated edges. Given the high feature-to-sample ratio (13,312 edges for N = 225), this network-based constraint is critical for dimensionality reduction.

On average, 320.3 edges were selected per fold (2.41% selection rate). Features were min–max scaled within each fold using MinMaxScaler, to prevent information leakage. Two regression algorithms were evaluated: L2-regularized linear regression (lambda = 1–1,000) and linear support vector regression (svmR; lambda = 0–1,000). Hyperparameters were tuned in the inner loop, and the best-performing model (svmR) was retained for final evaluation.

Predictive performance was quantified using Pearson’s correlation coefficient (r) between predicted and observed F3 scores; explained variance (R²) served as a secondary metric. Statistical significance was assessed via 1,000 permutation tests, in which F3 scores were randomly shuffled to generate a null distribution (p < 0.05, family-wise error corrected). The final weighted matrix (predictive network) was constructed by averaging edge weights across all folds, capturing both selection frequency (range: 0 = never selected, 1 = always selected) and contribution to model performance. Model stability was quantified as the consistency of feature selection across folds via the Jaccard similarity coefficient. For svmR, mean stability was 0.493 (range: 0.36–0.593); for linear regression, 0.387 (range: 0.271–0.585). Analyses were implemented in MATLAB using a fixed random seed (42) for reproducibility.

### 2.9 Network-level inference

For network-level inference, each region in the predictive network was assigned to one of eight canonical brain networks (Yeo et al., 2011)—subcortical, limbic, visual, sensorimotor, ventral and dorsal attention, frontoparietal, and default mode—based on a precomputed mapping from Genc et al. (2024). To account for differences in edge count across network pairs, the mean absolute predictive weight per pair was computed and tested against a spatial null distribution by permuting node-to-network assignments (N = 5,000). P-values reflect the probability that permutation weights exceed or equal the observed value; *q*-values control the false discovery rate (FDR) across the 36 network pairs. Permutation-based confidence intervals were estimated from the alpha/2 and 1−alpha/2 quantiles of the null distribution. Standardized effect sizes are reported as z-scores: (observed prediction strength − mu permutation)/sigma permutation, where mu and sigma refer to the mean and standard deviation of the permutation-derived null distribution, respectively.

### 2.10 Mediation and moderation models

Subcortical connectivity (SubC), which exhibited the strongest predictive contribution for decoding musical training, was entered into separate structural equation modeling (SEM) and regression-based moderation analyses. To avoid circularity, the subcortical connectivity index (SubC) was computed from the original, pre-NBS-Predict log(FBC) matrices rather than from NBS-Predict-selected features. SubC was defined as the mean log(FBC) across all edges linking the 16 subcortical to the 148 cortical regions, yielding a single global subcortical–cortical connectivity measure per participant. Of the 225 participants, 3 lacked WAIS-IV data; the remaining subsample (N = 222) was used for mediation and moderation analyses. SEM was conducted using the *lavaan* R package with full information maximum likelihood (FIML); exogenous predictors were treated as fixed. Moderation used ordinary least squares (OLS) regression. Age, biological sex, and IQ were included as covariates in both models, given their documented associations with musical training (Corrigall & Schellenberg, 2015; Criscuolo et al., 2019; Swaminathan & Schellenberg, 2018; Theorell et al., 2015). All continuous variables were z-standardized prior to modeling.

For mediation, the model specified F3 → SubC (a-path) and SubC → MET (b-path), plus the direct path F3 → MET (c′-path), adjusting for covariates. The indirect effect (a × b) was quantified using bias-corrected and accelerated (BCa) bootstrapped 95% confidence intervals (5,000 resamples). Mediation was tested via a likelihood-ratio comparison of the full model to a nested model with the b-path removed (delta-df = 1). The moderation analysis tested the F3 x SubC interaction term in the OLS model: MET ~ F3 + SubC + F3 × SubC + age + biological sex + IQ. Heteroskedasticity-consistent (HC3) robust standard errors assessed the interaction term. Simple slopes of F3 predicting MET were evaluated at -1 SD, mean, and +1 SD of SubC. Model comparison used the change in explained variance (delta-R²) between models with and without the interaction.

To separate SubC variance associated with training versus aptitude, edge-wise partial correlations were computed between each subcortical–cortical connection (log(FBC)) and F3 (controlling for MET, age, biological sex, and IQ). Edges were categorized based on the significance of *positive* partial associations (p < 0.05, uncorrected): training-associated (p_F3 < 0.05 and p_MET ≥ 0.05), aptitude-associated (p_MET < 0.05 and p_F3 ≥ 0.05), and shared (p_F3 < 0.05 and p_MET < 0.05). Negative partial associations were excluded to maintain directional consistency with the aggregate SubC index. For each participant, log(FBC) was averaged across edges within each category to derive three participant-level indices: SubC-training, SubC-aptitude, and SubC-shared. Mediation and moderation were then repeated substituting SubC with each index. Because this procedure constructs composite indices rather than making edge-level inferential claims, p-values served as feature-selection heuristic; robustness was evaluated via sensitivity analyses.

## 3 Results

### 3.1 Sample characteristics and analytical approach

**Fig. 1.**
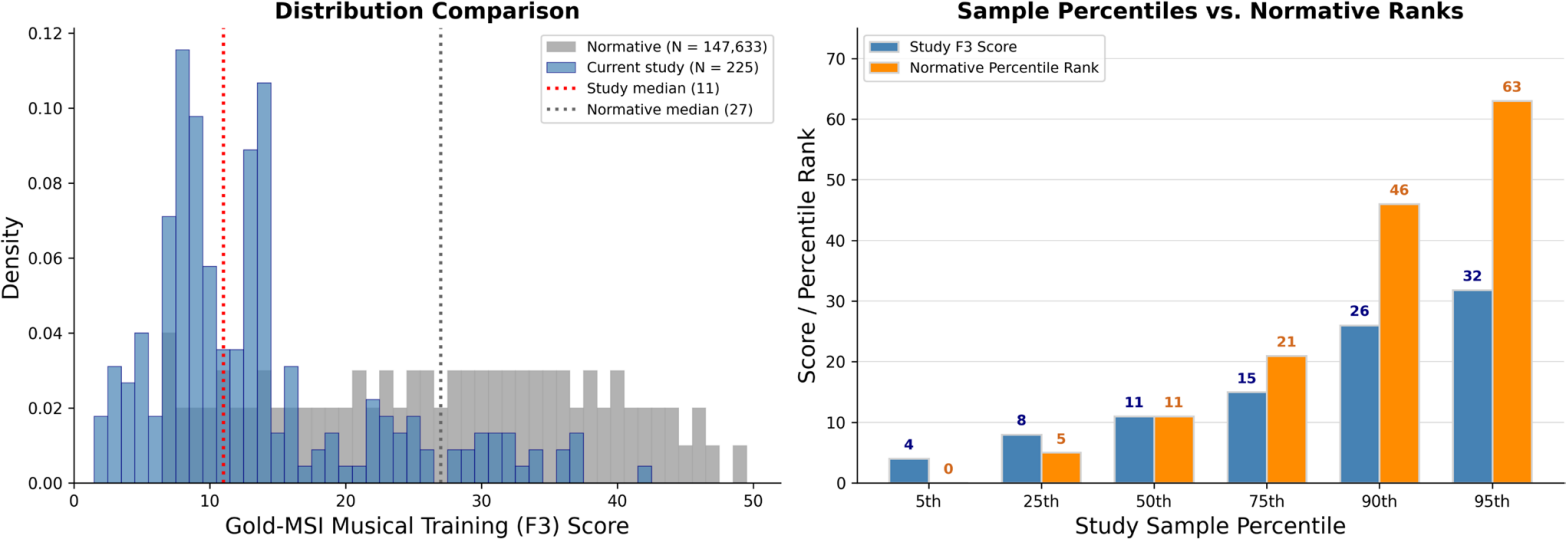
Distribution comparison of the Gold-MSI F3 scores between the current study and normative Gold-MSI data (Müllensiefen et al., 2014). Left panel: Overlaid histograms showing F3 score distributions for the current study (blue, N = 225) and normative sample (gray, N = 147,633). Dotted vertical lines mark the study median (11, red) and normative median (27, gray). Right panel: Comparison of sample percentiles (blue bars: actual F3 scores at each percentile within the study) against their normative percentile ranks (orange bars: where those scores fall in the general population). This comparison confirms that the sample predominantly comprises individuals with little or no formal musical training, with minimal representation of highly trained individuals.

Participants (N = 225) completed the Goldsmiths Musical Sophistication Index (Gold-MSI) (Müllensiefen et al., 2013) and the Musical Ear Test (MET) (Wallentin et al., 2010)—a standardized assessment of musical perceptual discrimination in rhythm and melody—alongside cognitive tests from the Wechsler Adult Intelligence Scale (WAIS-IV) (Lichtenberger & Kaufman, 2009). Most of our participants were non-specialists, reporting little or no formal musical training (Gold-MSI F3 scores: median = 11, interquartile range = 8–15, min–max range = 2–42). That corresponds to the 11th, 5th–21st, and <1st–90th percentiles (lower bounds) of the normative Gold-MSI Musical Training F3 distribution in the general population (N = 147,633) (Müllensiefen et al., 2014) (Fig. 1; Supplementary Fig. S2; Supplementary Table S2).

**Fig. 2.**
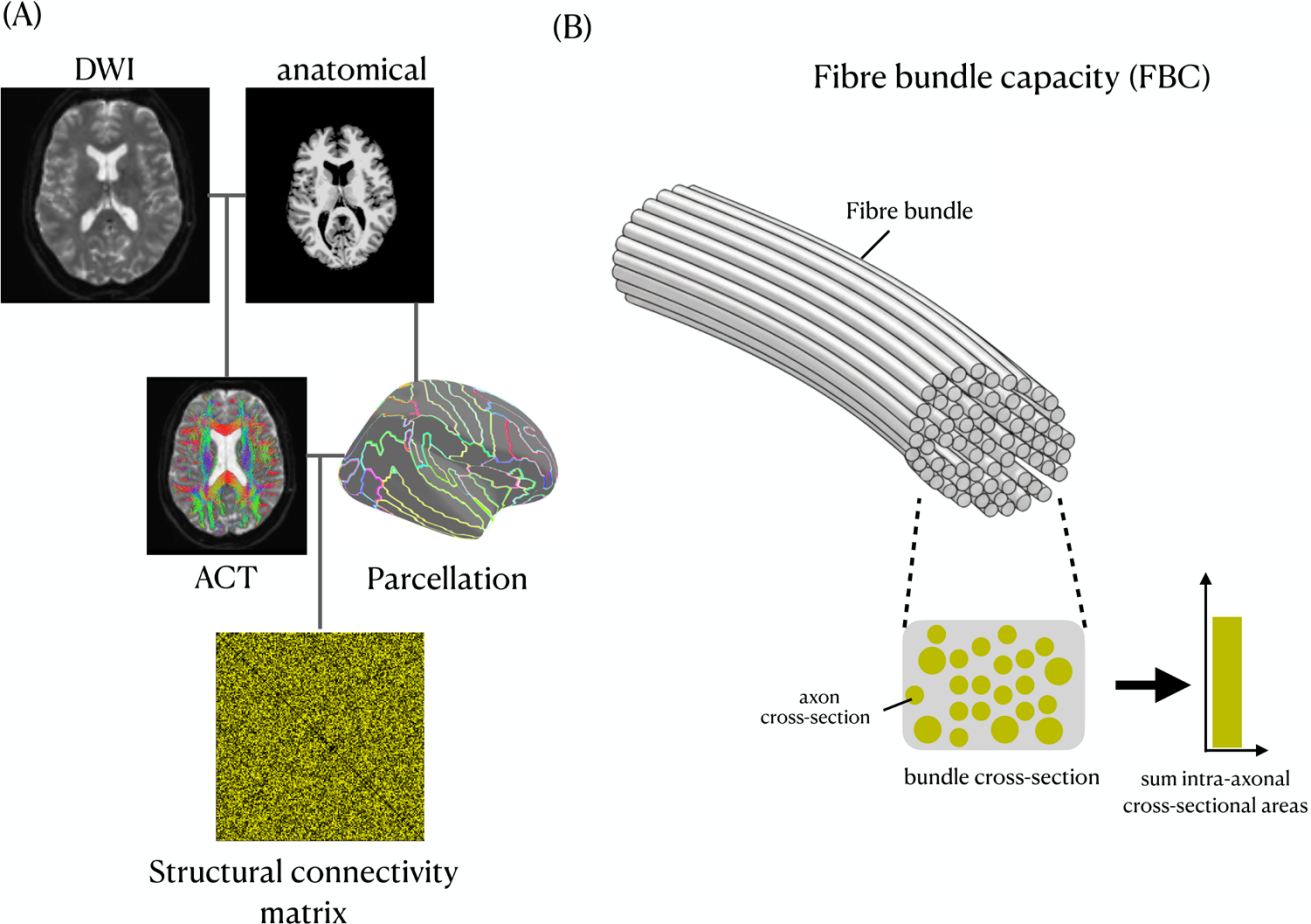
Flowchart of white-matter network construction. (A) Diffusion-weighted imaging (DWI) and synthetic T1-weighted anatomical images were used to construct whole-brain probabilistic anatomically constrained tractograms (ACT) of 10 million streamlines. The structural connectivity matrices were generated from these tractograms by integration with Destrieux cortical parcellation (74 cortical homologs) and FSL-based subcortical segmentation (8 subcortical homologs, excluding brainstem). Each edge of the structural connectivity matrix (164 x 164) represents fiber bundle capacity (FBC). (B) Visual depiction of the FBC metric. The FBC is defined as the sum of the cross-sectional areas of axons in the fiber bundle. This figure takes inspiration from Smith et al. (2022).

**Fig. 3.**
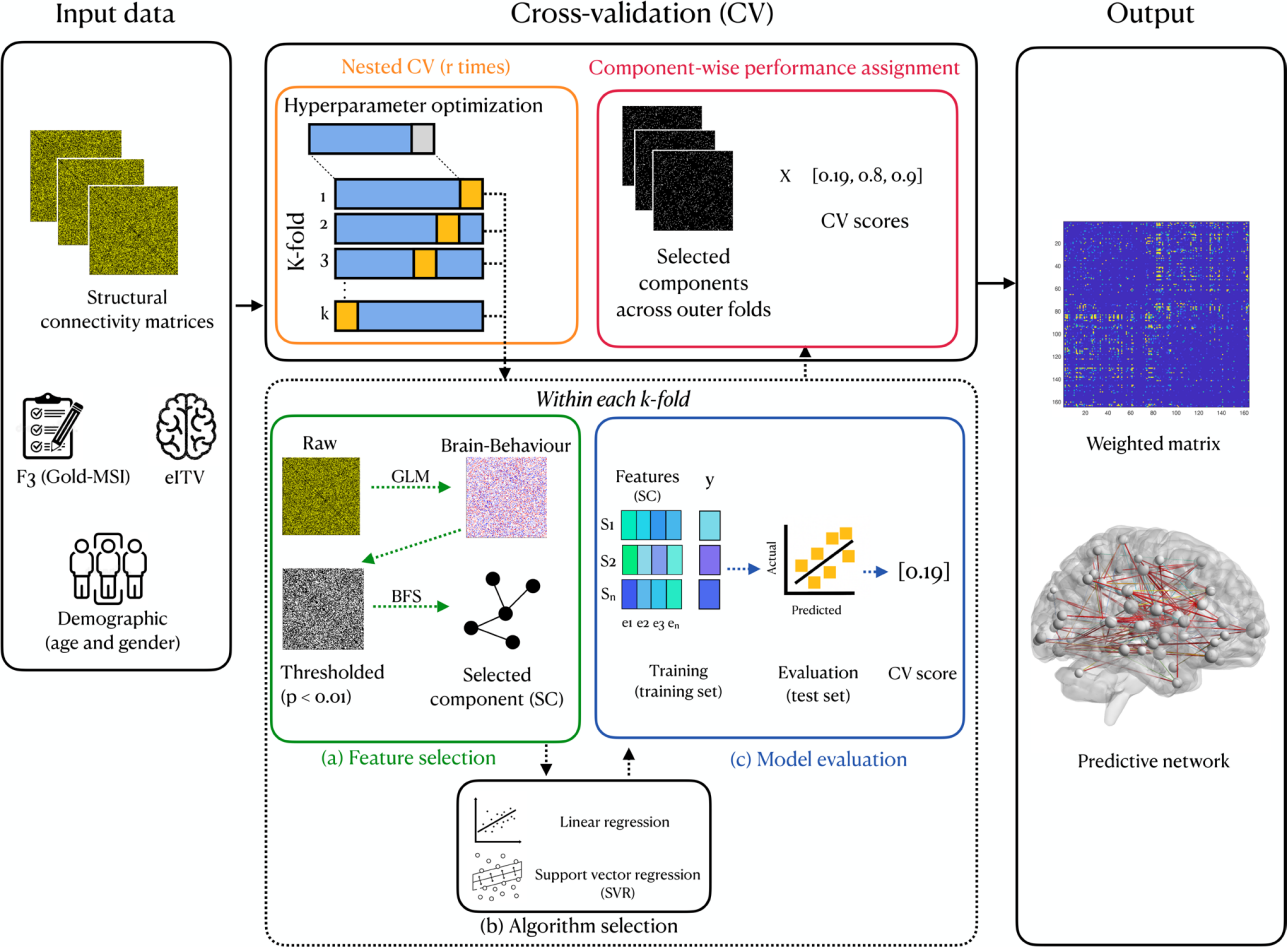
Schematic of the network-based statistics prediction (NBS-Predict) pipeline. Structural connectivity matrices, alongside demographic variables (age, biological sex), estimated intracranial volume (eITV), and Gold-MSI’s Musical Training Factor 3 (Gold-MSI F3) served as input data (N = 225). This algorithm implements a repeated (r = 10), nested 10-fold cross-validation structure with feature selection, scaling, and hyperparameter tuning occurring inside training folds of the inner loop. Specifically, steps include (i) an outer loop that performs suprathreshold edge selection (p < 0.01), selection of connected components through a breadth-first-search algorithm (BFS) (feature selection), algorithm selection, and model evaluation on the held-out test fold; (ii) an inner loop allowing for hyperparameter optimization. Across outer folds, connected components selected during feature selection are weighted by the model’s out-of-sample cross-validation (CV) scores. The output is a weighted adjacency matrix computed as the average of the adjacency matrices of the selected components across all outer folds. The weight assigned to each edge reflects both its frequency of inclusion across outer folds and the corresponding prediction performance. Statistical significance is assessed via 1,000 label-shuffled permutations.

For each participant, an anatomical image underwent parcellation using the Destrieux cortical atlas (Destrieux et al., 2010) (74 homologous regions) and subcortical segmentation (8 homologous structures) (Fischl, 2012), from which fiber bundle capacity (FBC)-weighted structural connectivity matrices were constructed (Fig. 2). These matrices were log-transformed and entered into the NBS-Predict pipeline (Fig. 3), with Gold-MSI F3 as the outcome variable, controlling for age, biological sex, and estimated intracranial volume (eITV). Prediction models (L2-regularized linear regression and linear support vector regression) were evaluated using repeated nested 10-fold cross-validation, with statistical significance assessed via nonparametric permutation testing. To examine which networks mainly carry predictive information, weights were aggregated within and between canonical brain networks (Yeo et al., 2011), with significance assessed through network-level permutation testing. Full analytical details, including structural equation modeling (SEM) and ordinary least squares (OLS) moderation specifications, are provided in Materials and Methods.

### 3.2 Whole-brain structural connectivity predicts musical training levels

**Fig. 4.**
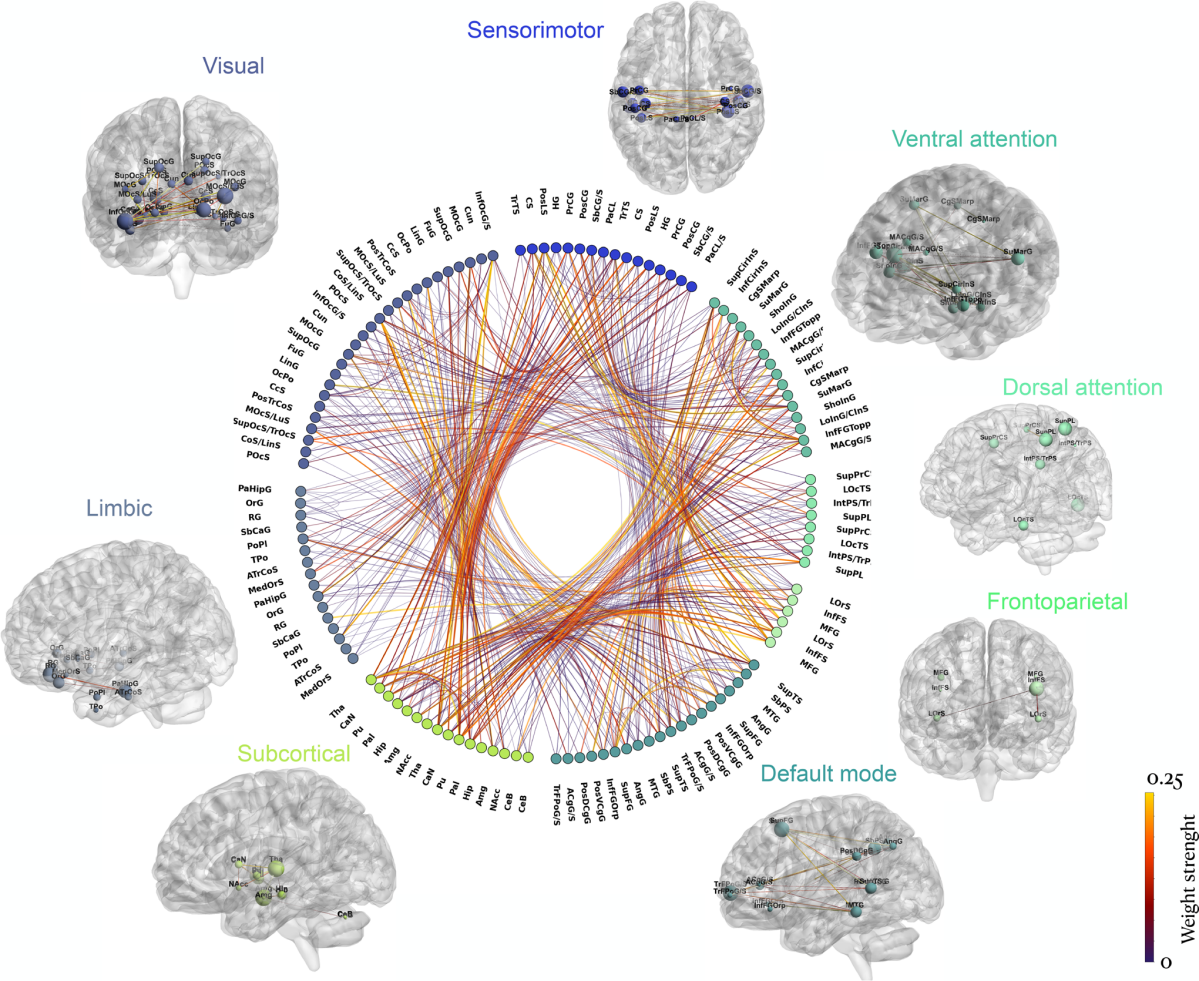
Main results of NBS-Predict using the support vector regression (svmR) algorithm. The weighted network is plotted on a circular connectogram, with nodes grouped by network (referenced in the accompanying glass brain). Edge color reflects predictive weight, with warmer hues indicating the connections with stronger predictive contribution (range: 0.0–0.25). The figure shows two prominent network pairs with less sparse bundles (i.e., highest predictive contributions): subcortical–sensorimotor and subcortical–ventral attention circuits.

Whole-brain structural connectivity, quantified by FBC, significantly predicted individual differences in musical training (Gold-MSI F3) (Table 1). Linear support vector regression (svmR) achieved the highest predictive performance, with a mean cross-validated Pearson’s coefficient of r = 0.261 between predicted and observed scores, explaining approximately 7% of the variance. This result was statistically significant after 1,000 nonparametric permutations (95% CI [0.253, 0.269]; *R^2^* = 0.068; p_perm_ = 0.003). The linear regression model (LinReg) yielded numerically lower performance, though still significant (r = 0.227, 95% CI [0.211, 0.244], *R^2^* = 0.051; p_perm_ = 0.003). Figure 4 displays the output of the best-performing model (svmR). Between-network connections constituted 86% of non-zero predictive edges, indicating that the signal was dominated by long-range white-matter tracts.

**Table 1.** Prediction performance of regression-based machine learning algorithms used to predict musical training scores (Gold-MSI F3) from structural connectivity.

Rank (by performance)	Algorithm	Repeated CV (r) μ_r_	Repeated CV (r) σ_r_	Stability	Permutation score	Permutation p-value
1	svmR	0.261	0.013	0.493	0.261	0.003
2	LinReg	0.227	0.026	0.387	0.240	0.003

*Note:* Pearson’s correlation coefficient (r) was used as a performance metric. Repeated CV (r) μ represents the mean correlation coefficient across 10 repetitions of 10-fold cross-validation (100 train–test partitions). Repeated CV (r) σ is the standard deviation of the cross-validation performance scores across the 10 repetitions. Model stability was quantified as the consistency of feature selection across folds, computed via the Jaccard similarity coefficient between selected edge sets. The permutation score denotes the observed test statistic against which the null (permuted) scores were compared.

### 3.3 Subcortical–sensorimotor circuits carry the strongest predictive signal

**Fig. 5.**
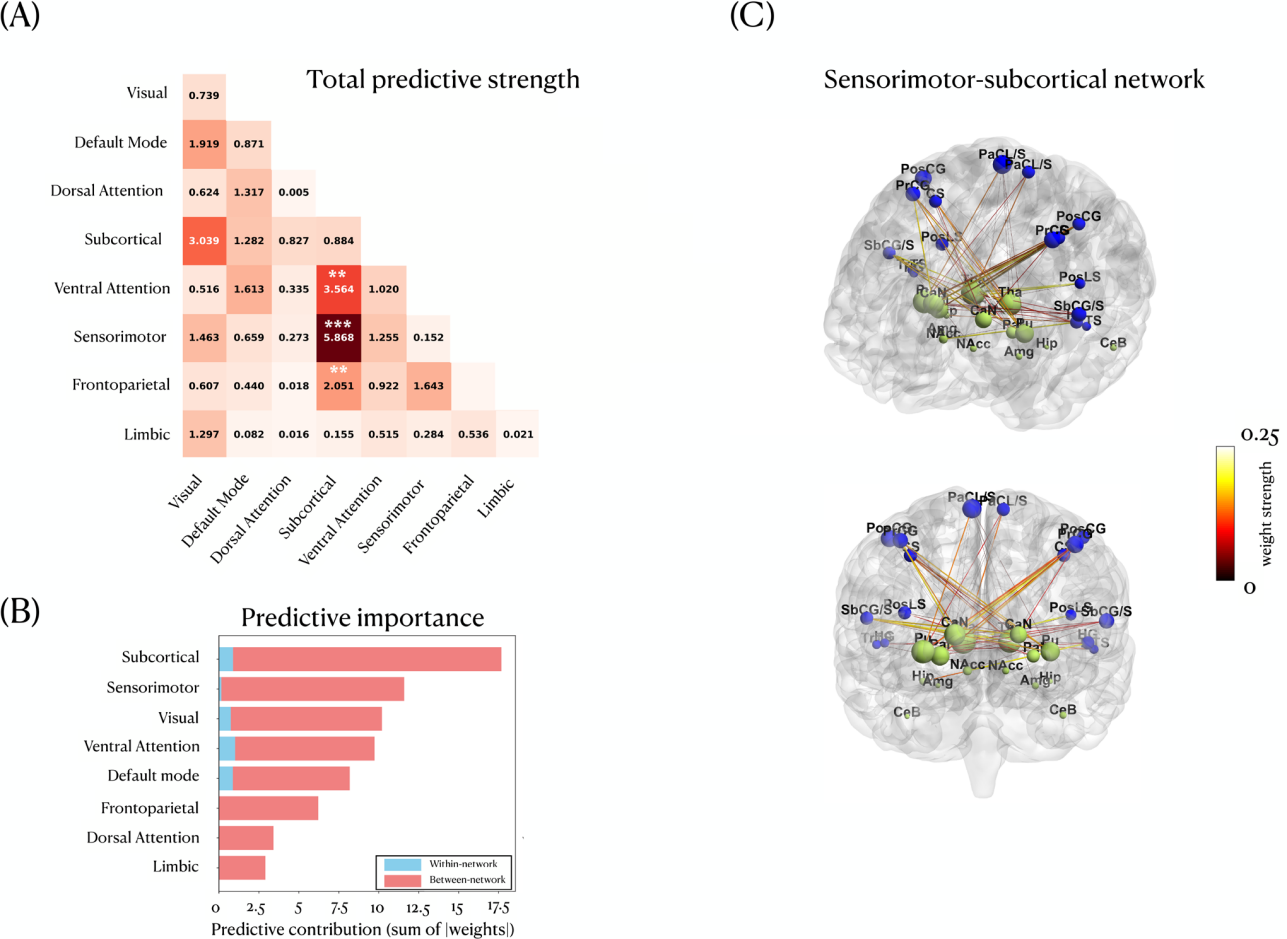
Network-based predictive contribution to musical training scores (Gold-MSI F3). Structural connectivity between subcortical regions and sensorimotor, ventral attention, and frontoparietal networks emerged as the strongest contributors to the prediction of F3. (A) Heatmap showing the predictive contribution of between- and within-network connectivity to F3. Each cell reflects the total predictive strength, computed as the sum of absolute edge weights connecting each pair of networks (between-network) or within a single network (within-network). Darker red indicates stronger predictive contribution. Asterisks mark FDR-corrected significance (** q < 0.01; *** q < 0.001). (B) Horizontal bar plot displaying total predictive contributions of each network, partitioned into within-network (blue) and between-network (red) components. Networks are rank-ordered by total contribution. Higher values reflect networks whose connectivity patterns more strongly predict individual differences in F3. (C) 3D brain rendering of the most predictive subnetwork: subcortical–sensorimotor connectivity. Edge thickness and color reflect predictive weight magnitude (range: 0.01–0.25), with thicker, brighter edges indicating stronger contributions.

**Fig. 6.**
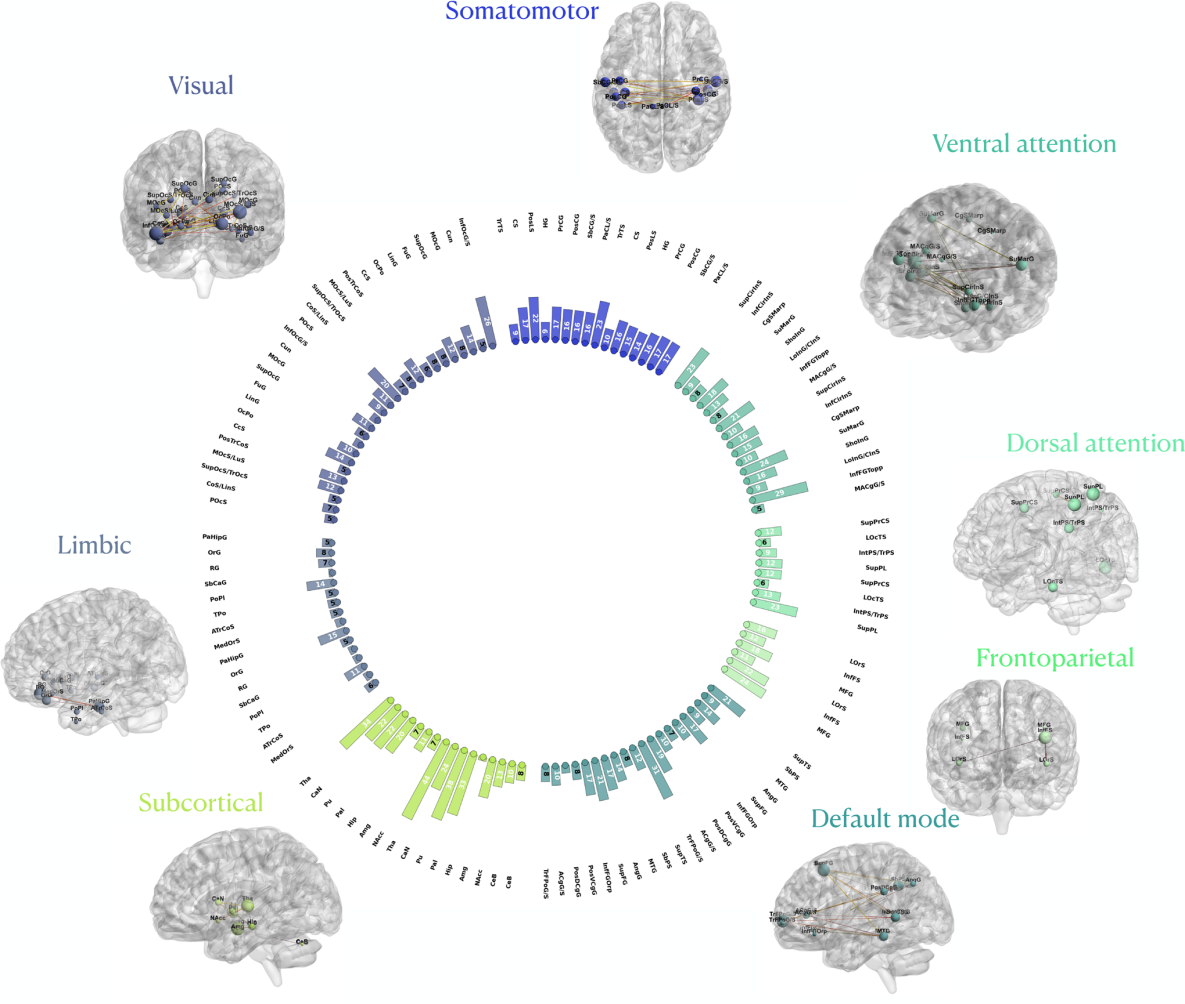
Node degree distribution in the predictive network. Circular connectogram displaying the degree (number of connections) for each brain region in the NBS-Predict output network. Nodes are organized by network (each represented in a glass brain). Right thalamus, right putamen, and right pallidum in the subcortical network showed the highest number of predictive connections.

To localize this predictive signal, we aggregated edge weights within and between eight canonical brain networks (Yeo et al., 2011). Permutation testing across 36 network pairs, with false discovery rate (FDR) correction (q = 0.01), revealed three significant pairs (Fig. 5A): subcortical–sensorimotor (N = 190 edges with non-zero weights; 95% CI [0.001, 0.0101]; q < 0.001; permutation-based effect size z = 8.075; Fig. 5C), subcortical–ventral attention (N = 211 edges with non-zero weights; 95% CI [0.001, 0.009]; q = 0.009; z = 4.04), and subcortical–frontoparietal (N = 65 edges with non-zero weights; 95% CI [0.0003, 0.013]; q = 0.009; z = 4.834). The subcortical network emerged as the single strongest contributor (Fig. 5B). At the nodal level, subcortical structures (right thalamus, right putamen, and right pallidum) showed the highest number of predictive connections (Fig. 6).

### 3.4 Musical training is positively associated with perceptual ability

Having localized the predictive signal to subcortical–sensorimotor circuits, we next examined the behavioral association between training and perceptual ability used in the mediation and moderation models. A partial correlation controlling for age and biological sex (N = 225) showed a small but significant positive association between Gold-MSI F3 scores and total MET scores (r = 0.15; p = 0.02) (Supplementary Fig. S5). This association was further supported by significant partial correlations between MET scores and objective training items from the F3 subscale (all p < 0.001, FDR corrected) (Supplementary Table S3; Supplementary Fig. S6). In participants with 0 years of formal training (N = 88), these items reflect untutored musical learning and practice.

### 3.5 Subcortical connectivity moderates the training–perception relationship

**Fig. 7.**
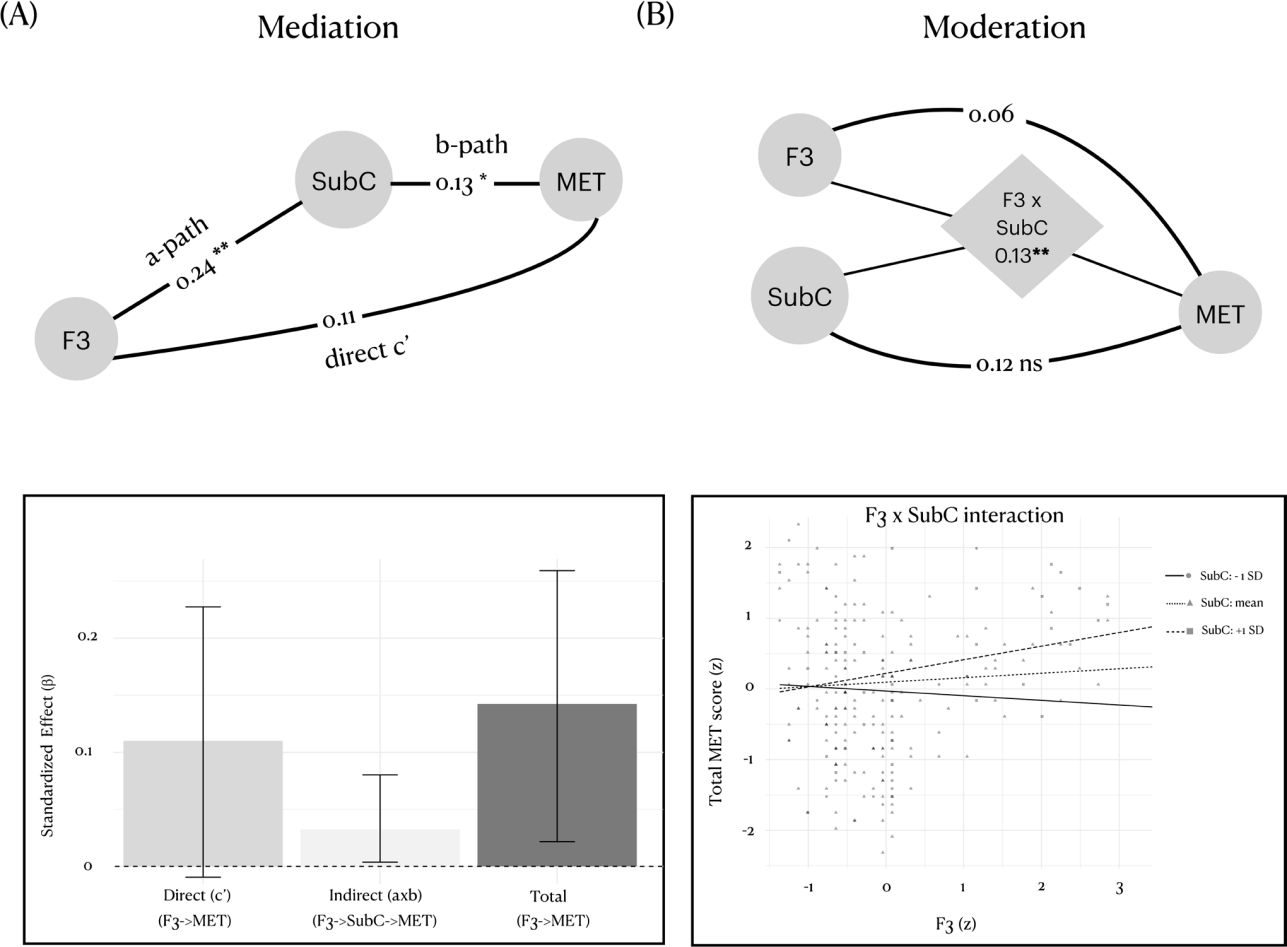
Cross-sectional mediation and moderation analyses (N = 222). These models characterize conditional associations among Gold-MSI Musical Training (F3), subcortical–cortical connectivity (SubC), and MET performance. Links in the diagrams do not establish temporal or causal pathways. (A) Mediation analysis: top, path diagram with standardized coefficients (β); bottom, standardized direct (c′), indirect (a×b), and total effects with 95% bootstrap confidence intervals (n = 5,000). (B) Moderation analysis: top, path diagram with standardized coefficients (β), including the interaction term (F3 × SubC); bottom, moderation plot showing predicted F3–MET slopes at three SubC levels (−1 SD, mean, +1 SD) derived from the continuous interaction model. Observed data points are binned into SubC tertiles for visualization only. Covariates (age, biological sex, and IQ) were not included in the figure. Abbreviations: F3, musical training scores; SubC, subcortical connectivity; MET, Musical Ear Test (total score).

We next tested whether subcortical connectivity (SubC) mediated the association between musical training and perceptual skill, while controlling for age, biological sex, and IQ (N = 222; 3 participants lacked WAIS-IV data). The bootstrapped indirect effect was small but statistically detectable (F3 → SubC → MET: a × b: β = 0.032, bias-corrected and accelerated 95% CI [0.004, 0.080]), indicating a conditional association consistent with the specified path model (Fig. 7A; Table 2). This indirect path accounted for approximately 23% of a small total effect (total β = 0.142). A nested likelihood-ratio test provided marginal statistical support for the SubC → MET path (Δχ²(1) = 4.221, p = 0.040), although the improvement in fit was small.

**Table 2.** Standardized coefficients (β), 95% confidence intervals, and p-values for mediation model paths linking musical training (Gold-MSI F3), subcortical connectivity (SubC), and perceptual ability (MET), controlling for age, biological sex, and IQ (N = 222).

Path	Nodes connected	β (std.)	SE	95% CI	*p*-value
a	F3 -→ SubC	0.246	0.077	[0.09, 0.397]	0.001 **
b	SubC → MET	0.131	0.062	[0.008, 0.255]	0.036*
c’	F3 → MET (direct)	0.11	0.059	[-0.009, 0.227]	0.065
ab	Indirect effect	0.032	0.018	[0.003, 0.08]	Bootstrap CI used for inference
c	Total effect	0.142	0.060	[0.0217, 0.259]	0.01 *

Significance thresholds: *p ≤ 0.05, **p ≤ 0.01.

Moderation analysis revealed a significant positive F3 × SubC interaction on MET performance (β = 0.128, p = 0.019) (Fig. 7B; Table 3). Simple-slope tests showed that the F3–MET association was significant only at high levels of SubC (+1 SD: β = 0.192, p = 0.004), but not at low (−1 SD: β = −0.066, p = 0.488) or mean levels (β = 0.063, p = 0.331). Including the interaction improved model fit, explaining a small amount of additional variance (R²: 0.167 → 0.188; ΔR² = 0.021), and the nested model comparison was significant (F(1, 215) = 5.545, p = 0.019). Inference was robust to heteroskedasticity (heteroskedasticity-consistent standard errors: p = 0.010). This pattern indicates that the cross-sectional training–perception association varied with subcortical–cortical connectivity strength.

**Table 3.** Standardized regression coefficients (β), standard errors (SE), test statistics (t or F), and p-values (two-tailed) for the moderation analysis testing the interaction between F3 and subcortical connectivity (SubC), controlling for age, biological sex, and IQ (N = 222).

Component	Estimate	SE	T-value	F-value	*p*-value
*Interaction model*					
F3	0.063	0.067	0.942		0.346
SubC	0.125	0.063	1.96		0.05
Interaction term (F3 × SubC)	0.128	0.054	2.354		0.019
*Conditional effects (simple slopes)*					
Low SubC (–1 SD)	-0.06	0.095	-0.69		0.488
Mean SubC (0)	0.063	0.065	0.97		0.331
High SubC (+1 SD)	0.192	0.066	2.9		0.004**
*Model comparison*					
R² (Base Model)	0.167				
R² (Interaction Model)	0.188				
ΔR²	0.021			F(1, 215) = 5.545	0.019*

The table includes the base model, the interaction model, conditional effects (simple slopes) at three levels of SC–SM, and nested model comparison statistics. Significance thresholds: *p ≤ 0.05, **p ≤ 0.01.

### 3.6 Exploratory edge-level decomposition of the SubC index

To examine whether the aggregate SubC index contained different classes of edges, some preferentially associated with Gold-MSI Musical Training, some with MET, and some associated with both measures, we performed an exploratory edge-level decomposition using partial correlations (uncorrected p < 0.05 threshold; see Methods). Given the high false-discovery risk, this decomposition was intended as a heuristic summary of association patterns rather than edge-level biological inference.

Of 1,696 subcortical–cortical edges, 123 (7.25%) were F3-specific (SubC-training), 68 (4.01%) were MET-specific (SubC-aptitude), and 19 (1.12%) were significant for both (SubC-shared) (Supplementary Fig. S7). The remaining 1,486 edges (87.62%) did not survive the statistical threshold and were discarded from further analyses. We averaged log(FBC) within each edge class to create participant-level indices and repeated the mediation and moderation analyses using these exploratory indices. The shared edge index showed the largest indirect association (beta = 0.078), whereas the F3-associated edge index showed the largest interaction term (beta = 0.196) (Table 4). In a competing-interactions model, only the F3-associated edge index retained a statistically detectable interaction term (beta = 0.130, p = 0.0079).

**Table 4.** Exploratory decomposition of SubC in training-related (SubC-training), aptitude-related (SubC-aptitude), and shared-related connectivity (SubC-shared), with mediation and moderation results (N = 222).

Edge class (index)	Number of edges (% of 1,696)	Mediation: indirect effect (BCa 95% CI)	p	Moderation: interaction β (HC3 SE)	p	Competing model: interaction β (HC3 SE)	p
**SubC-training**	123 (7.25%)	0.024 (-0.023, 0.080)	0.354	0.196 (0.050)	0.00011	0.13 (0.048)	0.0079
**SubC-aptitude**	68 (4.01%)	0.042 (-0.034, 0.120)	0.276	-0.053 (0.055)	0.337	-0.096 (0.066)	0.149
**SubC-shared**	19 (1.12%)	0.078 (0.039, 0134)	0.00096	0.133 (0.059)	0.0258	-	-

## 4 Discussion

Our findings indicate that individual differences in white-matter architecture, quantified via a structural connectivity metric proportional to intra-axonal cross-sectional area (Fiber Bundle Capacity, FBC), are linked with musical training and statistically moderate the strength of the association between training and music-perceptual skills. This finding complements prior cortico-centric and expert–novice frameworks of musical expertise by showing that subcortical–sensorimotor connectivity carries the strongest predictive information regarding graded variation in musical training among non-specialists.

Our regression-based framework differs in two respects from previous machine learning (ML) approaches to musical expertise, which have predominantly relied on task-based (Ribeiro & Thomaz, 2019; Saari et al., 2018) and resting-state functional neurophysiology (EEG/MEG) (C. Klein et al., 2016) to classify musicians versus non-musicians. First, functional measures are optimized to capture transient, state-dependent activity rather than neuroanatomical features that reflect experience-dependent adaptations, which are better captured by structural data (Hyde et al., 2009a; Scholz et al., 2009; Zatorre et al., 2012). Trait prediction from structural connectomes exhibits substantially higher replicability than prediction of transient states (Kotikalapudi et al., 2025). Second, binary classification neglects the continuous distribution of musical experience across individuals. Formal training and untutored learning represent end points on a spectrum that varies in context, style, and goals (Folkestad, 2006; Veblen, 2018)—nuances that binary classification inherently neglects, precluding subject-level predictions along a graded continuum (Kanai & Rees, 2011). Also, categorizing musicians and non-musicians relies on arbitrary definitions, such as the number of years of formal music instruction (Ilari & Habibi, 2023), which is inconsistent across studies (Daly & Hall, 2018; J. D. Zhang et al., 2020), and in its essence does not capture the complexity of the musical experiences of individuals (Zentner & Gingras, 2019). By modeling training as a continuous variable, we capture the multifaceted nature of musical engagement (Folkestad, 2006; Putkinen et al., 2013). This approach extends the focus from features that distinguish experts from novices (Penhune, 2019) to those predicting individual training history and engagement, moving closer to understanding how brain architecture relates to learning potential in non-specialist adults within the general population (Cogo-Moreira & Lamont, 2018; Müllensiefen et al., 2014).

The observed effect size for the significant association between structural connectivity and musical training (R² = 0.068, r = 0.26) is modest but expected given the multifactorial nature of musical expertise, shaped by neurogenetic, cognitive, environmental, and experiential factors (Mosing et al., 2014; Ullén et al., 2016). It is consistent with other well-powered, cross-validated studies predicting individual differences in behavioral and cognitive measures from brain connectivity (Dubois et al., 2018; Serin et al., 2021; Shafiei et al., 2024). In-sample variance is virtually always an overly optimistic estimate of true predictive accuracy because model parameters are optimized to the training data, thus fitting sample-specific noise (Yarkoni & Westfall, 2017). When the ratio of predictors to sample size is high, this inflation is substantial, with simulations showing that 50 subjects and 20 weak predictors yield an in-sample variance explained of 0.45 but an out-of-sample variance of only 0.02 (Yarkoni & Westfall, 2017). This is why the small- and within-sample correlation studies that dominate the musical-expertise literature (Abdul-Kareem et al., 2011; Bengtsson et al., 2005; Chaddock-Heyman et al., 2021; Foster & Zatorre, 2010; Groussard et al., 2014; Seither-Preisler et al., 2014; Sluming et al., 2002; Steele et al., 2013) tend to inflate effect sizes and often fail to generalize to new samples of individuals (Poldrack et al., 2017). Such studies of musical expertise have offered an initial, exploratory view of potential brain–behavior associations (Gabrieli et al., 2015; Poldrack et al., 2017), but their effects would be expected to shrink substantially when tested in independent samples (Button et al., 2013). Cross-validation combined with a large sample yields more realistic estimates of generalizability, and our pipeline was built to this end: NBS-Predict reduces dimensionality through network-based feature selection (retaining only the largest connected component) and nested cross-validation, with regularization further constraining overfitting (Serin et al., 2021). The resulting estimate falls within the range that large-scale benchmarking associates with reliable replication: structural connectome predictions replicate well at moderate sample sizes (N < 300) once variance explained exceeds approximately 5%, whereas associations below 2% generally require much larger samples (N > 400) and have limited practical utility (Kotikalapudi et al., 2025). In these studies, replicable effects across phenotypes have been demonstrated with discovery samples averaging N = 171–228 (N = 150 for trait-like measures) (Kotikalapudi et al., 2025). Our effect (≈7%) and sample (N = 225) fall within this replicable range.

Subcortical–sensorimotor connectivity emerged as the strongest predictor of musical training scores, suggesting that subcortical pathways may play a more prominent role in music-related plasticity than previously recognized. Structural connectivity studies comparing musicians and non-musicians remain scarce in the literature, and heterogeneous in their findings (Moore et al., 2014). Most report training-related differences in cortical regions, notably the arcuate fasciculus (Brown et al., 2015; Herholz et al., 2016; Herholz & Zatorre, 2012; Pando-Naude et al., 2021) and corpus callosum (Bengtsson et al., 2005; Schlaug et al., 1995; Schmithorst & Wilke, 2002), particularly for early trained individuals (Bengtsson et al., 2005; Schlaug et al., 1995). Evidence for subcortical–cortical sensorimotor pathways is limited, with descending motor pathways examined as primary tracts of interest in only a few studies (Engel et al., 2014; Imfeld et al., 2009; Rüber et al., 2015) and inconsistent FA directions reported: lower in some (Imfeld et al., 2009; Schmithorst & Wilke, 2002) and higher in others (Bengtsson et al., 2005; Han et al., 2009; Rüber et al., 2015). Andrews et al. (2021) is the only study to have reported an effect in the striatal pathway (the sagittal striatum). Thalamo-cortical and cortico-striatal pathways—the white-matter projections through which the thalamus projects to sensorimotor cortices and through which sensorimotor cortices project to the striatum, respectively (Behrens et al., 2003; Verstynen et al., 2012)—have not been examined as primary targets in any prior diffusion MRI study on musical expertise. Our findings suggest that, in adults with predominantly little or no formal musical training, accumulated musical experience is most strongly associated with these subcortical–cortical projections. These pathways form components of cortico-basal ganglia–thalamo–cortical loops implicated in musical processes, including millisecond-precision timing in rhythm perception, beat tracking, and synchronization (Cannon & Patel, 2021; Patel & Iversen, 2014; Schwartze et al., 2011; Toiviainen et al., 2020): core abilities underpinning musical listening expertise across the general (non-specialist) population. Importantly, these circuits are not music specific; they also support procedural learning and temporal processing across domains, including motor sequence learning and predictive timing (Colder, 2015; Wolff et al., 2022). These circuits are phylogenetically conserved and strongly heritable (Milardi et al., 2019), potentially providing a stable anatomical scaffold that may be shaped by training for music skill acquisition in humans (Dehaene & Cohen, 2007).

Our results show that subcortical–cortical pathways of the predictive network have a right-dominant profile, with subcortical hubs (right thalamus, putamen, and pallidum) exhibiting the highest degree. Existing evidence on the lateralization of descending motor and internal-capsule pathways comes almost exclusively from studies of expert musicians (Criscuolo et al., 2022), leaving it unclear whether similar patterns hold in individuals with moderate, little, or no formal training. Some diffusion studies of expert musicians have described right-sided or right-weighted effects. Bengtsson et al. (2005) found that childhood practice correlated with FA in the bilateral posterior internal capsule, whereas the difference between musicians and controls was significant only in the right posterior internal capsule. Han et al. (2009) similarly reported higher FA in the right posterior limb of the internal capsule in pianists. Rüber et al. (2015) further showed that FA in right-hemispheric pyramidal and alternate motor fiber tracts was higher in both keyboard and string players, whereas left-hemisphere effects were present only in keyboard players, suggesting that left-sided adaptations may depend more strongly on instrument-specific bimanual demands. Giacosa et al. (2019) also reported predominantly right-hemisphere differences in descending motor pathways, attributing them to non-dominant hand training in right-handed musicians. However, other studies have reported bilateral changes in corticospinal or internal capsule in musicians (Imfeld et al., 2009; Schmithorst & Wilke, 2002), indicating that the interpretation of training-induced lateralization effects remains unsettled. Importantly, these studies mainly concerned expert musicians and focused on the effects of extensive motor practice, whereas the Gold-MSI Musical Training (F3) score in the present sample puts less weight on motor practice and more weight on informal musical experience and self-referential musicianship. The present finding should, therefore, be interpreted as a descriptive feature of the predictive network for musical training in non-experts, rather than as evidence that instrumental musical practice produces right-lateralized subcortical organization.

FBC offers interpretive advantages over traditional connectivity measures. Streamline count is unreliable due to tractography reconstruction biases, while fractional anisotropy is difficult to interpret in regions with crossing fibers, which are predominant throughout the brain (~90% of white-matter voxels) (Jeurissen et al., 2013; Jones et al., 2013; Maier-Hein et al., 2017; Yeh et al., 2016). Both these metrics are now considered semi-quantitative and of limited biological interpretability (Smith et al., 2022). By contrast, using more advanced, high-resolution anatomical (Weiskopf et al., 2013) and diffusion images (Descoteaux, 2015) to build connectomes, FBC indexes total intra-axonal cross-sectional area of a pathway (Smith et al., 2022), providing a more directly interpretable estimate of its capacity (“bandwidth”) for fast, parallel axonal communication. Higher FBC can reflect either more axons (greater bandwidth) or larger-diameter axons (faster conduction) (Perge et al., 2012), and axon caliber is itself modulated by neuronal activity (Almeida & Lyons, 2017) and by skill learning (Wang et al., 2014). These observations raise the possibility that training-associated FBC patterns partly reflect activity-dependent structural adaptations in subcortical–sensorimotor circuits, although directionality cannot be established from the present cross-sectional data. FBC is weighted toward intra-axonal architecture rather than myelin (Smith et al., 2022), but this specificity is graded, not absolute, and is stronger at high b-value shells (b ≥ 3,000 s/mm²; Tahedl et al., 2025). Because our acquisition included the highest shell of b = 2,500 s/mm², together with lower shells, we cannot fully exclude a contribution of concurrent myelination changes to the differences reported here. Such changes, an increasingly recognized mechanism of activity-dependent plasticity (Bacmeister et al., 2020; Bonetto et al., 2021; Fields, 2015; McKenzie et al., 2014; Sampaio-Baptista et al., 2013), were not targeted by our methodology and remain uncharacterized here. We, therefore, interpret our findings as predominantly reflecting intra-axonal architecture, while acknowledging a possible role of myelin. For time-critical neural computations in music learning and perception, conduction velocity or bandwidth may be limiting factors (Luo et al., 2017). Our results underscore the importance of white-matter bandwidth in distributed networks, a property that mass-univariate diffusion analyses (e.g., diffusion tensor imaging and fixel-based analyses) (Ashburner & Friston, 2000; Raffelt et al., 2017) may not adequately characterize. Connectivity weights with a clear microstructural basis, such as FBC, should improve predictive network models of expertise that generalize across individuals and domains.

Whether expertise reflects accumulated experience, predispositions, or an interaction of both remains a long-debated issue that is still unresolved (Ericsson et al., 1993; Galton, 1891; Mosing et al., 2014; Tan et al., 2014). While our cross-sectional data cannot settle this question or establish causal mechanisms, it can help characterize the associative structure of the subcortical–sensorimotor signal identified by the prediction model. The mediation analysis indicated a small, but statistically reliable, indirect association linking training, subcortical–cortical connectivity, and auditory perceptual performance, although cross-sectional mediation cannot verify the temporal ordering assumed by the path model. The moderation analysis, based on a single aggregate subcortical–cortical connectivity index (SubC), indicated that the association between training and auditory perceptual ability varied with connectivity strength: a positive association between Gold-MSI Musical Training (F3) and MET performance was observed only at high connectivity levels, with no significant association at mean or low levels. In exploratory, uncorrected edge-level analyses probing whether this aggregate index was uniform, the F3-associated edge index showed the largest interaction term in the moderation model, whereas the shared-edge index showed the largest indirect association in the mediation model. Because these edge indices were defined using uncorrected thresholds, they represent heuristic summaries of association patterns rather than separable biological components. Together, these patterns point toward testable predictions for future work. Longitudinal or genetically informed designs could determine whether specific subcortical–cortical connections moderate skill acquisition over time, and whether the training-associated and shared components of the subcortical network reflect distinct developmental or experiential processes.

Our findings suggest a potential role for ML-derived structural biomarkers in personalized music education (Gabrieli et al., 2015; Zatorre, 2013) and music-based neurorehabilitation (Grau-Sánchez et al., 2020; Sihvonen et al., 2017). In other domains, structural biomarkers derived through cross-validation of well-sampled datasets have demonstrated predictive value for identifying differential training responses, from reading (Hoeft et al., 2007, 2011) to mathematics (Supekar et al., 2013), sometimes matching or exceeding traditional behavioral and cognitive predictors (Gabrieli et al., 2015). Similar prediction-focused approaches remain unexplored in the musical domain and could test whether training regimens could be tailored to learners with different connectivity profiles (Gabrieli et al., 2015). In rehabilitation, the circuits we identified overlap with those targeted by music-based interventions such as rhythmic auditory stimulation (RAS) and music-supported therapy (MST) (Sihvonen et al., 2017), where substantial heterogeneity in patient outcomes remains poorly explained (McCrary et al., 2022). The structural integrity of descending motor pathways, particularly the corticospinal tract, is already recognized as among the strongest predictors of stroke recovery (Lin et al., 2018; Lindenberg et al., 2010; Pinter et al., 2020). If replicated in patient samples using longitudinal designs, such biomarkers might help identify individuals most likely to benefit from specific training regimens. Given the modest effect size observed here, however, such applications remain speculative.

Three limitations warrant consideration. First, the present study differs from prior expert–novice research along several dimensions simultaneously: the use of continuous rather than binary modeling of musical training (Daly & Hall, 2018; Ilari & Habibi, 2023; J. D. Zhang et al., 2020), a predominantly non-specialist sample with little or no formal training levels (Correia et al., 2023), and FBC-weighted structural connectomes rather than the regional diffusion-tensor (Moore et al., 2014; Schmithorst & Wilke, 2002) or functional measures (Saari et al., 2018) employed in most previous studies. This prevents us from attributing the differences between our findings and the earlier literature to any single factor, or to their interaction. Applying the present continuous, whole-brain pipeline to participants spanning the full expertise range—including conservatory-trained musicians—would help isolate the contribution of sampling from those of analytical approach and connectivity measurement, and would clarify whether the moderating role of structural connectivity scales non-linearly or remains stable across expertise levels. Second, the cross-sectional design precludes causal inference; training-related connectivity differences may reflect experience-dependent plasticity (e.g., related to onset age of training) (Abraham, 2008), pre-existing predispositions that influence engagement, or their combined contribution. Longitudinal studies tracking training, skill, and connectivity over time, which remain rare in the field of music expertise (Hennessy et al., 2019; Hyde et al., 2009b), ideally complemented by genetic data, will be necessary to disentangle these contributions. Third, our sample comprised predominantly individuals with little or no formal training, limiting generalizability to the full expertise spectrum observed in the general population, which also includes specialists (Müllensiefen et al., 2014). Despite these constraints, the focus on non-specialists with little or no training enhances relevance to ordinary musical education, where most individuals have little or no history of formal musical lessons outside the general school curriculum for music, and it is useful to better disentangle accumulated experience from pre-existing aptitude in neural signatures of expertise.

In conclusion, these results carry implications for both basic neuroscience and applied domains. At the theoretical level, our findings highlight subcortical–sensorimotor circuits as a key component of the structural correlates of musical expertise in “non-experts”, thereby extending the cortico-centric focus that has dominated the field of musical expertise to subcortical white matter. At the applied level, the observed moderating role of white-matter architecture suggests that structural connectivity profiles could eventually inform individualized approaches to music education and rehabilitation, pending replication in longitudinal and clinically diverse samples. More broadly, by showing that even modest, everyday musical engagement leaves detectable traces in subcortical white-matter organization, this work underscores the sensitivity of prediction-based connectomic approaches for studying experience-dependent plasticity across the full spectrum of human behavior.

## Supplementary Material

Supplementary Material

## Data Availability

Data cannot be shared publicly as it is part of an ongoing study, and thus considered unanonymized under Danish law, even if pseudonymized. Researchers who wish to access the data may contact Dr Kristian Sandberg (kristian.sandberg@cfin.au.dk) at The Center of Functionally Integrative Neuroscience and/or The Technology Transfer Office (TTO@au.dk) at Aarhus University, Denmark, to establish a data-sharing agreement. After permission has been given by the relevant ethics committee, data will be made available to the researchers for replication purposes. As the project is ongoing, sharing requests for other purposes will be evaluated on a case-by-case basis. Codes for reproducing diffusion-MRI analyses and results are available on GitHub: https://github.com/MassimoLumaca/neuroARC and https://github.com/MassimoLumaca/neuroTraining.
