# Supplementary material for: Decoding everyday levels of musical training from subcortical white-matter architecture
Source: Imaging Neurosci (Camb). 2026 Aug 4;4:IMAG.a.1325. doi: 10.1162/IMAG.a.1325 (PMC13440155; doi:10.1162/IMAG.a.1325)
Supplement: Supplementary file 1 [file IMAG.a.1325_supp.pdf]

Supplementary Material

Decoding everyday levels of musical training from subcortical white-matter architecture

Supplementary Figures

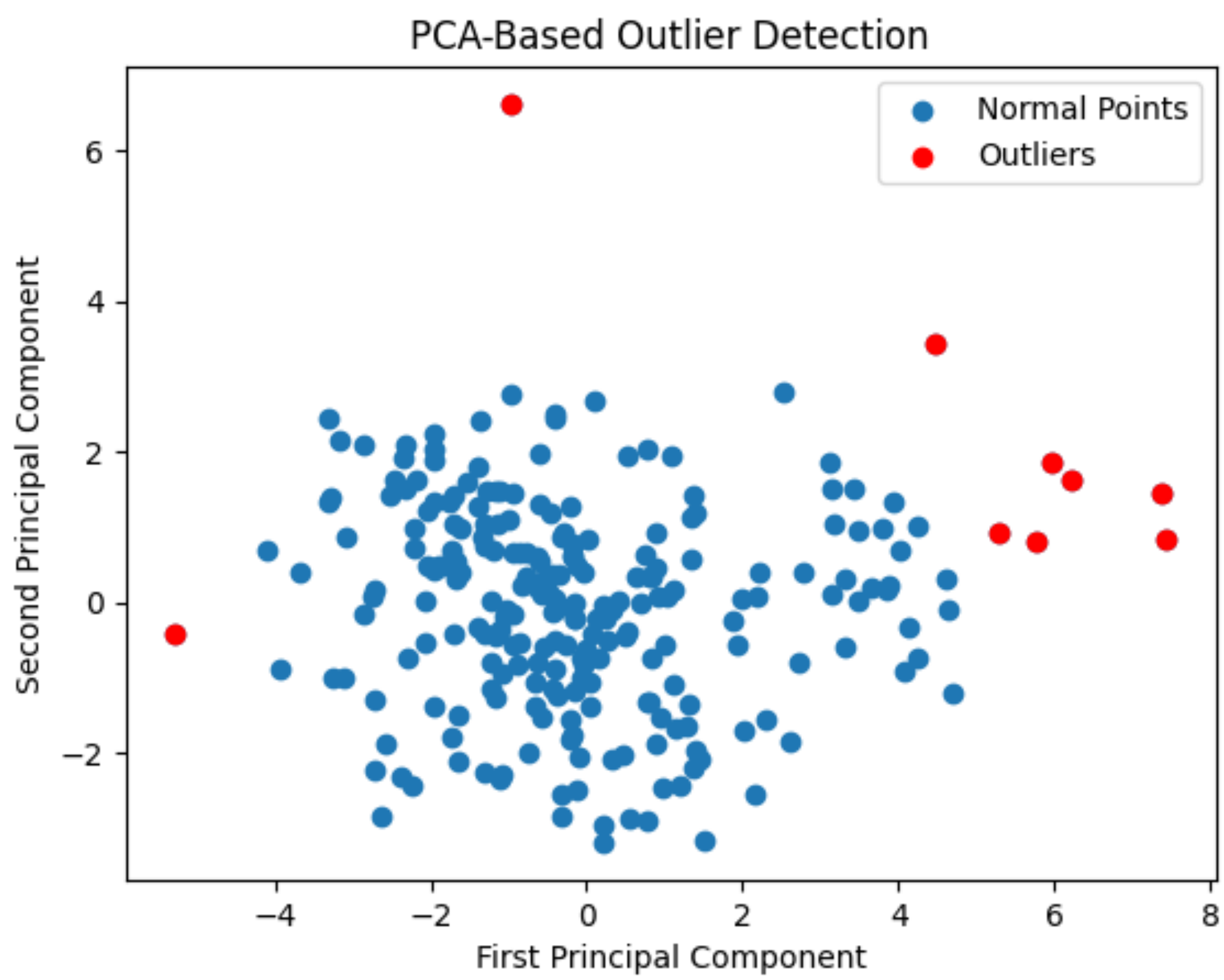

Supplementary Figure 1. Scores from the Musical Ear Test (MET) - encompassing total, rhythm, and melody - and Goldsmiths Musical Sophistication Index (Gold-MSI) subscales were standardized via z-score normalization to ensure equal weighting of variables (N=241). The resulting dataset was reoriented to align with principal axes of maximum variance. Outlier detection involved calculating Euclidean distances from the origin in the

PCA plane, with primary emphasis on the first two components. Points exceeding two standard deviations from the mean Euclidean distance (N=9) were designated as outliers. An additional seven participants were excluded due to failure in structural network reconstruction, resulting in a total of 225 participants comprising Gold-MSI and MET data.

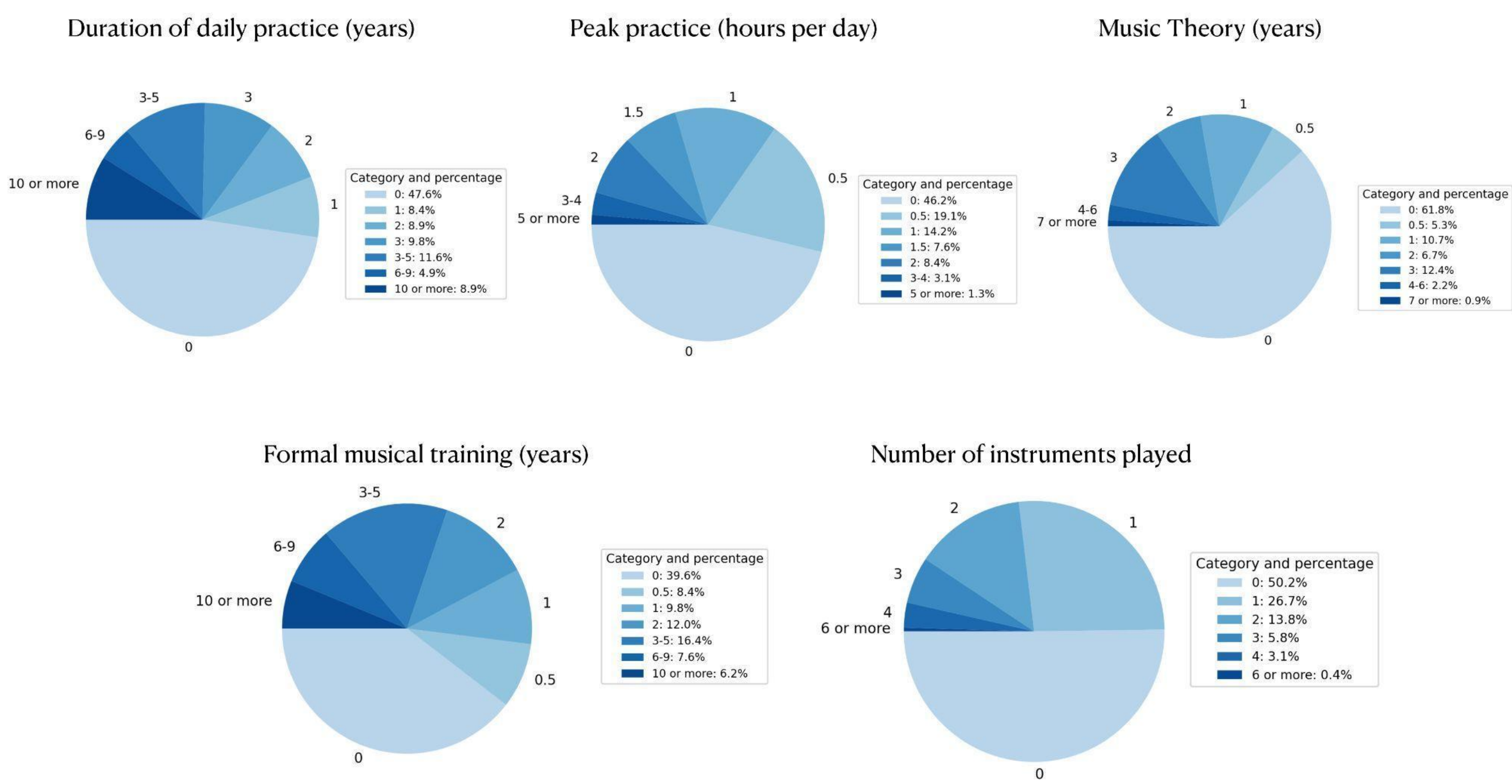

Supplementary Figure 2. Pie charts depicting the frequency of musical training duration and number of instruments played among participants across individual items the Gold-MSI Music Training subscale (N=225). Percentages in the legend indicate the proportion of participants categorized by the relative Gold-MSI Music Training item.

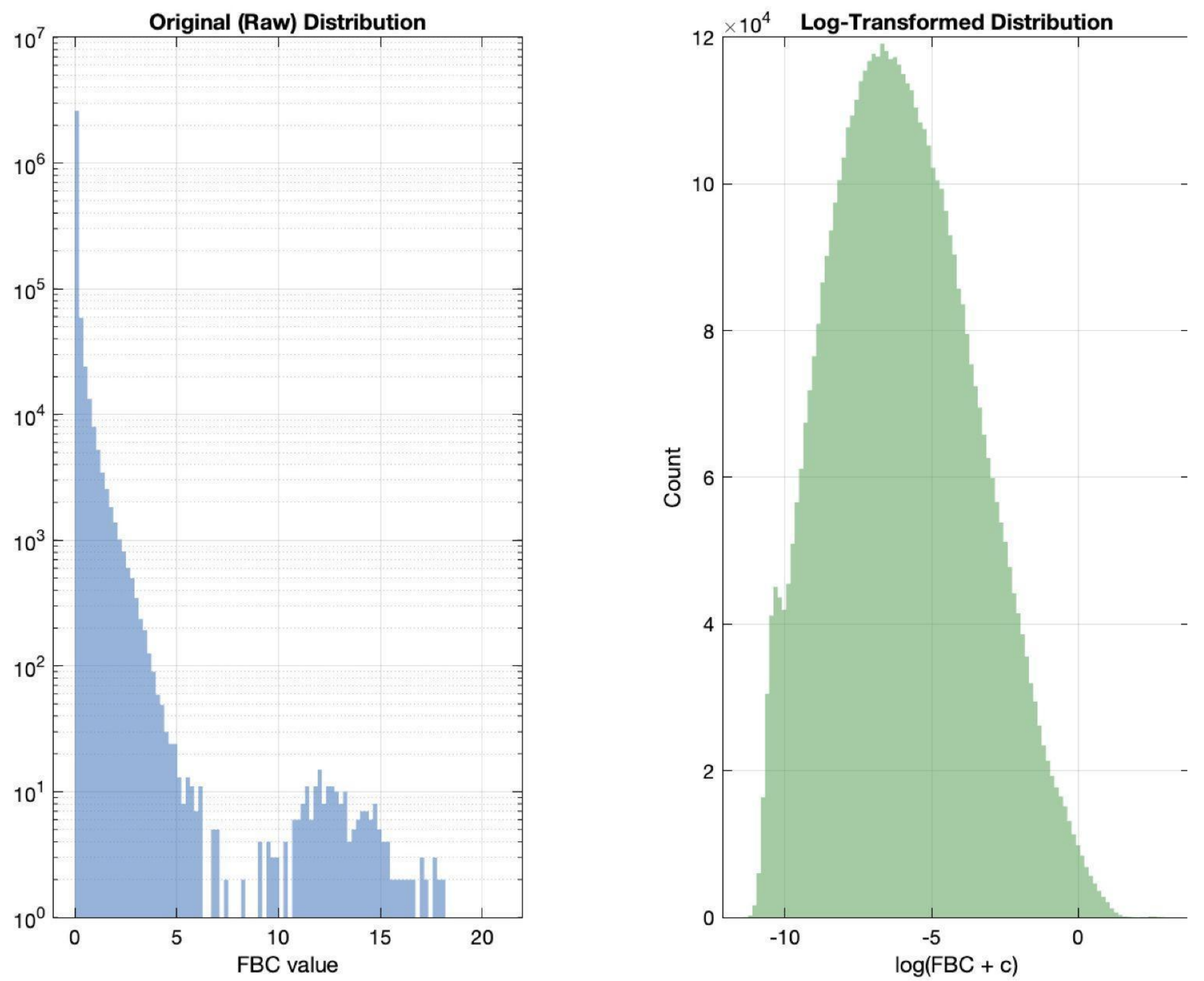

Supplementary Figure 3. Fibre bundle capacity (FBC) distribution before and after logarithmic transformation.

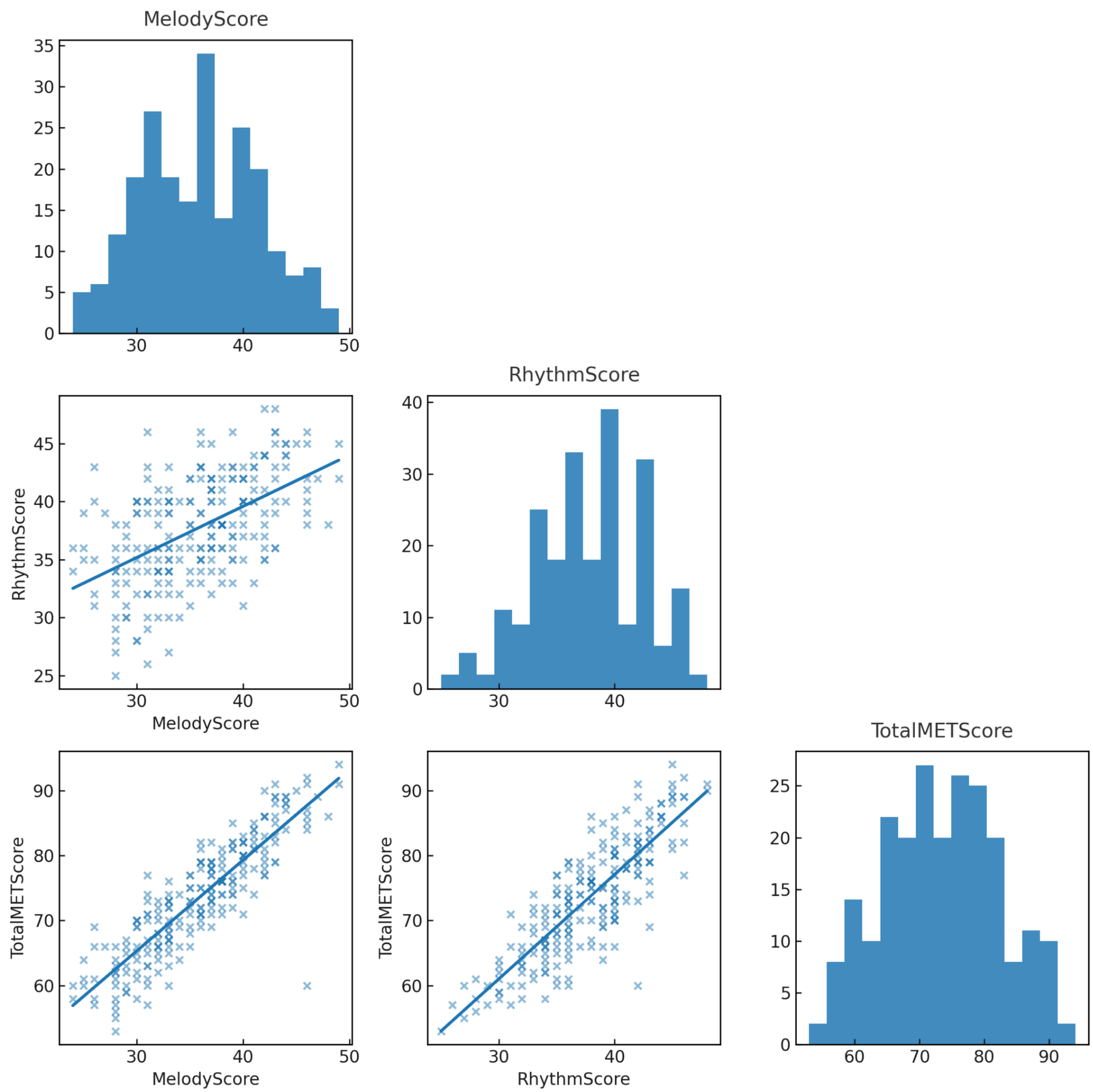

Supplementary Figure 4. Correlation matrix between MET rhythmic, melodic, and total scores. Each point in the scatterplot is a participant (N=225).

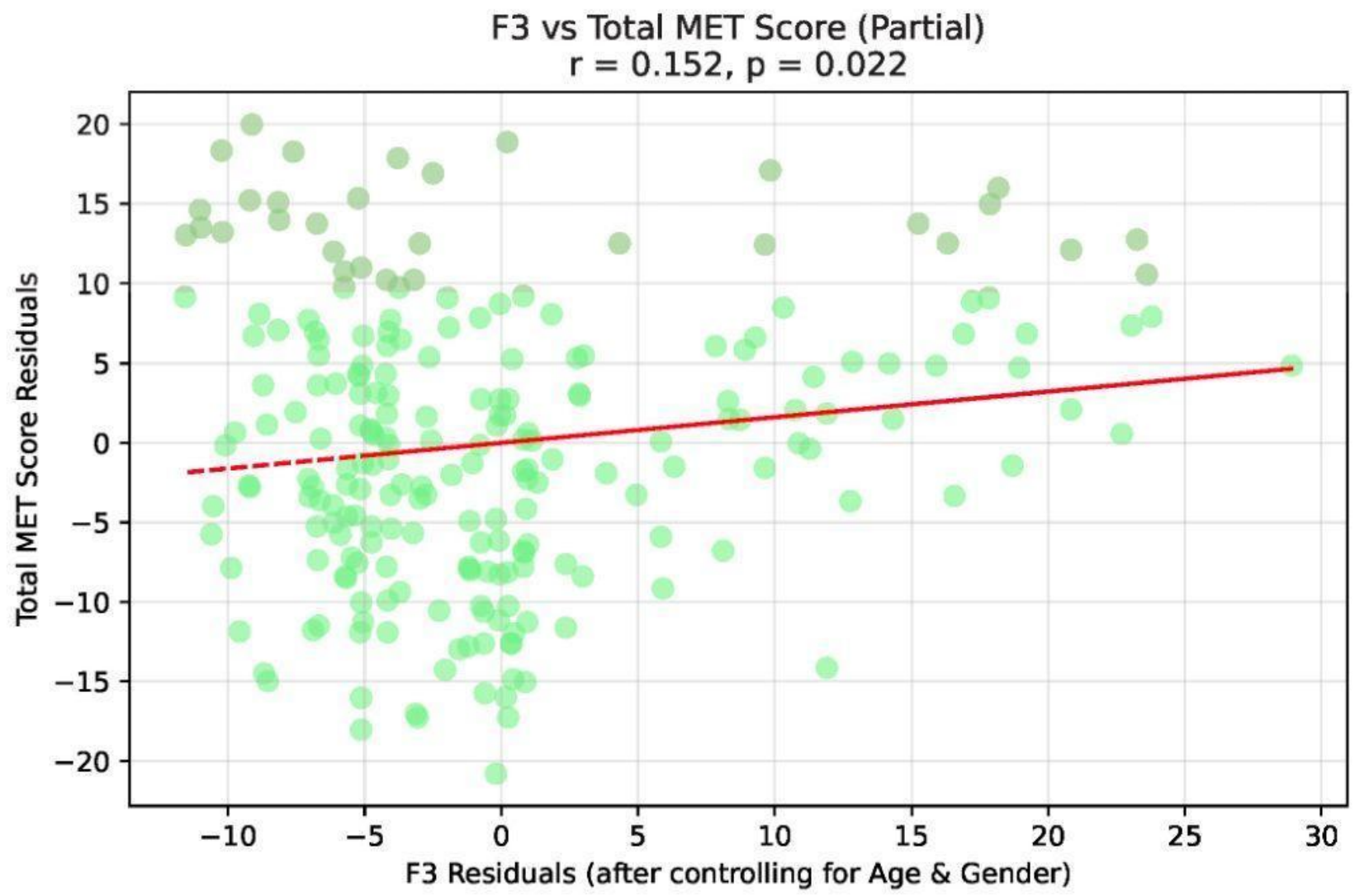

Supplementary Figure 5. Partial correlations between MET total scores and musical training (F3). Scatterplots show the association between residuals, after removing effects of covariates (age and biological sex). Each datapoint corresponds to one participant (N=225).

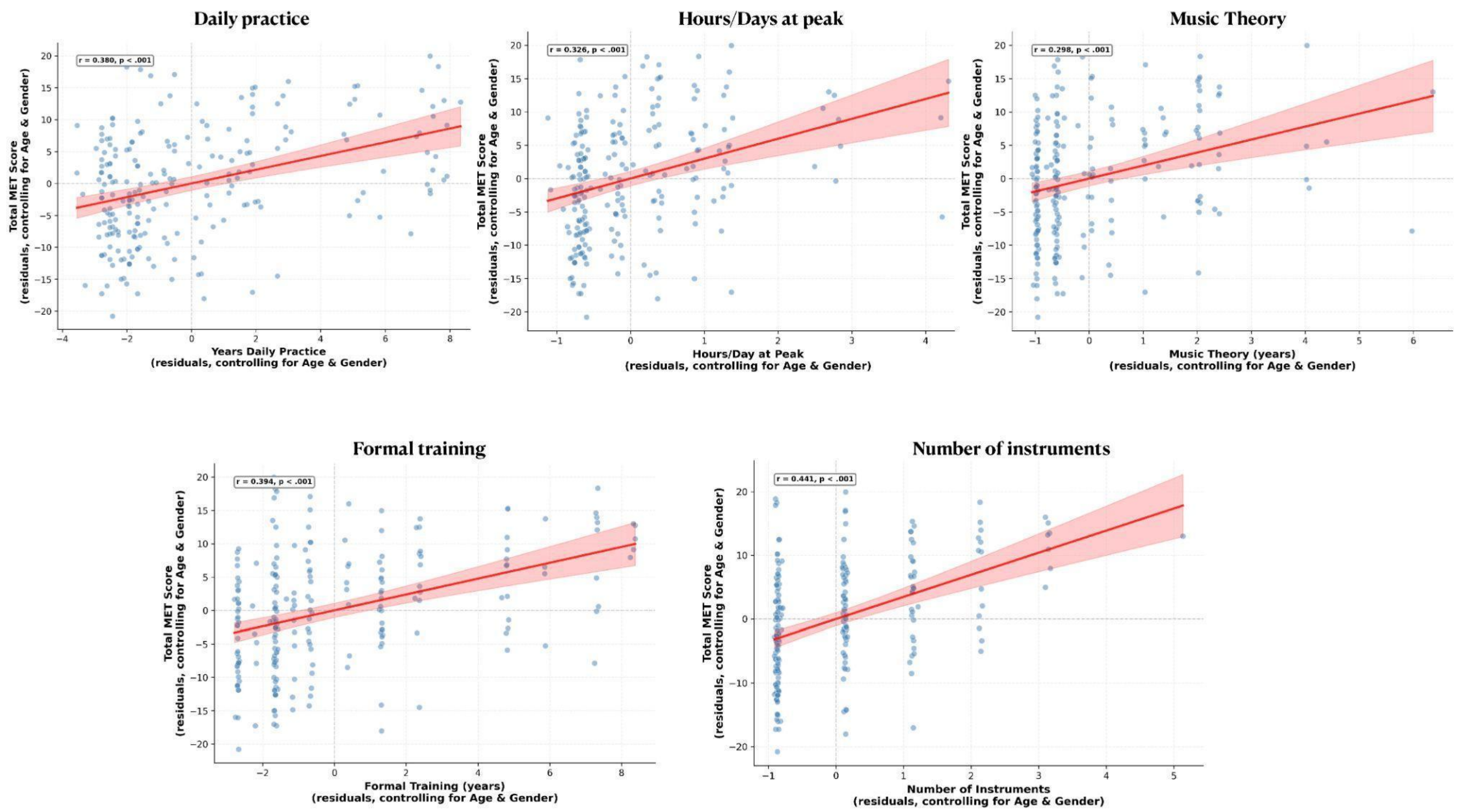

Supplementary Figure 6. Partial correlations between MET total scores and individual items from the Music Training subscale of the Gold-MSI. Scatterplots show the association between residuals, after removing effects of covariates (age and biological sex). Each datapoint corresponds to one participant (N=225).

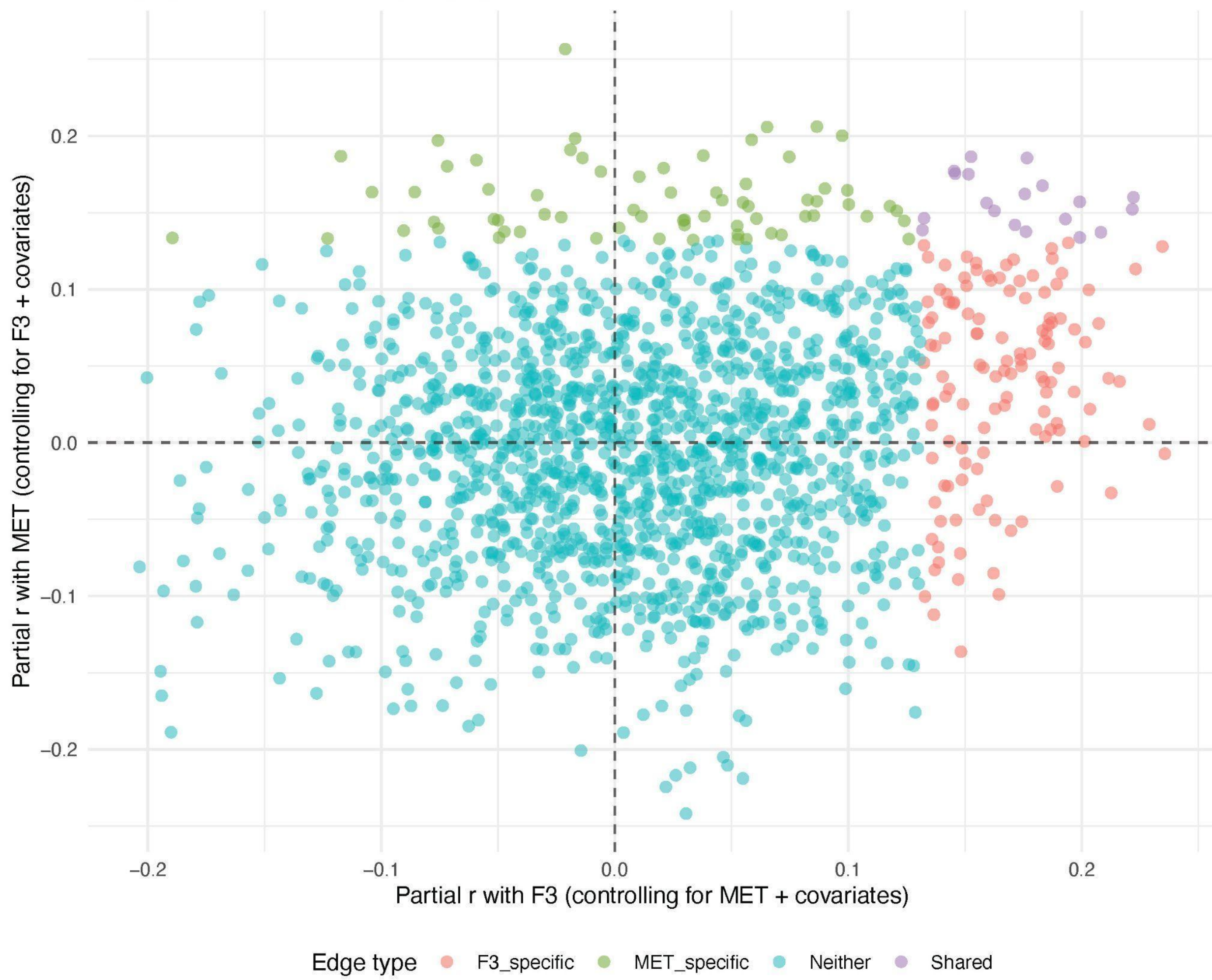

Supplementary Figure 7. Edge classification by partial correlations of SubC edges. Scatterplot showing, for each subcortical–cortical edge, the partial correlation with training (x-axis) and the partial correlation with aptitude (y-axis). Covariates include age, biological sex, and IQ from the WAIS-IV. Edges are colored by classification based on positive partial correlations at $p < 0.05$ (uncorrected): F3-specific (training-associated) edges are significant for F3 only (N=123), MET-specific (aptitude-associated) edges are significant for MET only (N=68), Shared edges are significant for both (N=19); Neither indicates edges not meeting these criteria (i.e., edges with negative partial correlations and $p > 0.05$). Each data point is one edge (N=1,696).

Supplementary Tables

Supplementary Table 1. Summary of reviews, meta-analyses, and experimental studies examining white-matter structural connectivity in relation to musical training. A dash (-) indicates that no tracts of that category were reported or showed no significant differences between groups.

#	Authors	Year	Group(s)	Structural connectivity analyses	Examined	Identified/ discussed: cortico-cortical tracts	Identified/discussed: cortico-subcortical tracts
1	Schlaug et al.	1995	mus vs nonmus	MRI morphometry	ROI (CC)	CC	-
2	Schmithorst & Wilke	2002	mus vs nonmus	VBA DTI	whole-brain white matter	CC, ILF	Corona radiata, IC
3	Bengtsson et al.	2005	mus vs nonmus	VBA DTI	whole-brain white matter	CC, AF	IC
4	Hyde et al.	2009	children (music training vs nonmusic) longitudinal	Deformation-based morphometry (DBM)	whole-brain	CC	-
5	Han et al.	2009	mus vs nonmus	VBA DTI	whole-brain white matter	-	IC
6	Imfeld et al.	2009	mus vs nonmus	DTI deterministic tractography	ROI (CST, SLF, UF, CC, IFO, ILF)	-	CST
7	Oechslin et al.	2009	absolute pitch vs relative pitch vs nonmus	DTI fibre tracking	ROI (SLF)	SLF	-
8	Wan & Schlaug	2010	Review	N/A	N/A	CC, AF	IC, CST
9	Halwani et al.	2011	singers vs instrumentalists vs nonmus	DTI probabilistic tractography	ROI (AF)	AF	-
10	Steele et al.	2013	Early trained mus vs late trained mus vs nonmus	TBSS DTI	whole-brain and post-hoc ROI analysis (CC)	AF, CC	-
11	Ruber et al.	2013	keyboard players vs string players vs nonmus	DTI probabilistic tractography	ROI (PT, aMF from M1, PMd, SMA)	-	PT, aMF

12	Engel et al.	2014	nonmus	TBSS DTI	Whole-brain white matter<br><br>ROI (CST, SLF, cerebellar peduncles)	SLF/AF	CST
13	Moore et al.	2014	Review	N/A	N/A	CC, AF, SLF	CST, IC, cerebellar peduncles (contradictory findings)
14	Schlaug	2015	Review	N/A	N/A	CC, AF, SLF	IC, CST (contradictory findings)
15	Elmer et al.	2016	mus vs nonmus	DTI probabilistic tractography	ROI (CC)	CC	-
16	Habibi et al.	2017	children (music vs sports vs controls)	DTI	ROI (CC)	CC	-
17	Vaquero et al.	2018	nonmus	DTI deterministic tractography	ROI (AF)	AF	-
18	Giacosa et al.	2019	dancers vs musicians vs controls	Multitensor probabilistic tractography	ROI (descending motor tracts)	-	Descending motor tracts
19	Loui et al.	2019	children (mus training vs nonmus training)	TBSS	ROI (SLF, CC)	SLF	-
20	Rajan et al.	2019	adults with different training levels	TBSS probabilistic tractography	Whole-brain white matter<br><br>post-hoc ROI (CC and corona radiata)	CC	Corona radiata
21	Andrews et al.	2021	lifelong professional mus (ages 20-67)	DTI	ROI (SLF, UF, sagittal striatum)	SLF, UF	Sagittal striatum
22	Li et al.	2021	mus vs nonmus	DTI deterministic tractography	ROI (AF, CST)	AF	-
23	Vaquero et al.	2021	nonmus	DTI deterministic tractography	ROI (AF)	AF	-
24	Criscuolo et al.	2022	ALE meta-analysis (mus vs nonmus)	WM-only ALE	N/A	-	IC, CST

* Abbreviations: **AF**, arcuate fasciculus; **aMF**, alternative motor fibres; **CC**, corpus callosum; **CST**, corticospinal tract; **IC**, internal capsule; **IFO**, inferior frontal-occipital fasciculus; **ILF**, inferior longitudinal fasciculus; **PT**, pyramidal tract; **SLF**, superior longitudinal fasciculus; **UF**, uncinate fasciculus

Supplementary Table 2. Descriptive statistics for Gold-MSI and MET subscales in the full sample (N=241).

	Mean	SD	Range
**Gold-MSI scores**			Score Range (theoretical max)
Music Training	14.04	9.28	2-47 (49)
General Sophistication	59.61	16.90	27-121 (126)
**MET**			Score Range (theoretical max)
Total	73.53	9.16	38-94 (104)
Melody	35.99	5.52	24-49 (52)
Rhythm	37.80	4.59	25-48 (52)

Gold-MSI: Music Training (amount of formal and informal musical training received); General Sophistication (general index of musical sophistication); MET: Melody and Rhythm scores were calculated from 52 trials. Total scores were calculated from 104 trials. In this table, we report the descriptive statistics of the raw scores.

Supplementary Table 3. Partial correlations between MET scores and individual items from the music training subscale of the Gold-MSI (after correcting for age and biological sex, N = 225). P-values are FDR-corrected.

	MET total		MET melody		MET rhythm	
**Gold-msi item**	r	p	r	p	r	p
Years of daily practice	0.38	<.001	0.39	<.001	0.268	<.001
Hours/Days	0.326	<.001	0.323	<.001	0.252	<.001

at peak						
Music theory (years)	0.298	<.001	0.308	<.001	0.247	<.001
Formal training (years)	0.394	<.001	0.382	<.001	0.314	<.001
Number of instruments	0.44	<.001	0.441	<.001	0.327	<.001
